# Supplementary material for: Improving public understanding of ‘overdiagnosis’ in England: a population survey assessing familiarity with possible terms for labelling the concept and perceptions of appropriate terminology
Source: BMJ Open. 2018 Jun 27;8(6):e021260. doi: 10.1136/bmjopen-2017-021260 (PMC6020944; doi:10.1136/bmjopen-2017-021260)
Supplement: Author's manuscript [file bmjopen-2017-021260.draft_revisions.pdf]

BMJ Open is committed to open peer review. As part of this commitment we make the peer review history of every article we publish publicly available.

When an article is published we post the peer reviewers' comments and the authors' responses online. We also post the versions of the paper that were used during peer review. These are the versions that the peer review comments apply to.

The versions of the paper that follow are the versions that were submitted during the peer review process. They are not the versions of record or the final published versions. They should not be cited or distributed as the published version of this manuscript.

BMJ Open is an open access journal and the full, final, typeset and author-corrected version of record of the manuscript is available on our site with no access controls, subscription charges or pay-per-view fees (http://bmjopen.bmj.com).

If you have any questions on BMJ Open's open peer review process please email info.bmjopen@bmj.com

BMJ Open

Improving public understanding of 'overdiagnosis': a population survey assessing familiarity with possible terms for labelling the concept and perceptions of appropriate terminology

Journal:	*BMJ Open*
Manuscript ID	bmjopen-2017-021260
Article Type:	Research
Date Submitted by the Author:	19-Dec-2017
Complete List of Authors:	Ghanouni, Alex; University College London, Research Department of Behavioural Science and Health<br>Renzi, Cristina; UCL, Research Department of Behavioural Science and Health<br>Waller, Jo; UCL, Research Department of Behavioural Science and Health
Keywords:	EPIDEMIOLOGY, PUBLIC HEALTH, Health policy < HEALTH SERVICES ADMINISTRATION & MANAGEMENT

SCHOLARONE™
Manuscripts

For peer review only

FULL TITLE PAGE

Title: Improving public understanding of ‘overdiagnosis’: a population survey assessing familiarity with possible terms for labelling the concept and perceptions of appropriate terminology

Manuscript type: Research article

Authors:

Alex Ghanouni[a] a.ghanouni@ucl.ac.uk

Cristina Renzi[a] c.renzi@ucl.ac.uk

Jo Waller[ab] j.waller@ucl.ac.uk | Tel: +44 (0)20 7679 5958 | Fax: +44 (0)20 7679 8354

Affiliations:

[a]Research Department of Behavioural Science and Health, University College London, Gower Street, London, WC1E 6BT, United Kingdom

[b]Corresponding author

Word count: Abstract: 245/300; Body: 2,178/4,000; References: 20; Tables or figures: 3

For peer review only

ABSTRACT

Objectives: Communicating the concept of ‘overdiagnosis’ to lay individuals is challenging, partly because the term itself is confusing. This study tested whether alternative descriptive labels may be more appropriate.

Design: Questionnaire preceded by a description of overdiagnosis.

Setting: Home-based, computer-assisted face-to-face survey.

Participants: 2,111 adults aged 18 to 70 years in England recruited using random location sampling by a survey company. Data from 1,888 participants were analysed after exclusions due to missing data.

Interventions: Participants were given one of two pieces of text describing overdiagnosis, allocated at random, adapted from NHS breast and prostate cancer screening leaflets.

Primary and secondary outcome measures: Main outcomes were which of several available terms (e.g. ‘overdetection’) participants stated they had previously encountered and which they endorsed as applicable labels for the concept as described. Demographics and previous exposure to screening information were also measured.

Results: 58.0% of participants had not encountered any of the available terms; 44.0% did not endorse any as applicable labels. No term was notably familiar; the proportion of participants stating they had previously encountered each term ranged from 15.9 to 28.3%. Each term was only endorsed as applicable by a minority of participants (range: 27.6% to 40.4%). Findings were similar for both pieces of information.

For peer review only

Conclusions: Familiarity with suggested terms for overdiagnosis and levels of endorsement were low and no clear alternative labels for the concept were identified, suggesting that changing terminology alone would do little to improve understanding. Explicit descriptions may be more effective.

Keywords: Decision Making; Surveys and Questionnaires; Early Detection of Cancer; Mass Screening; Medical Overuse

Strengths and limitations of this study

- This study assessed familiarity and perceived appropriateness of a broad range of possible terms for the concept of 'overdiagnosis' among a large, representative sample of the general public in England.
- The concept was described based on information that was widely used by the National Health Service in England, maintaining generalisability.
- The list of possible terms was not exhaustive; more suitable alternative labels may have been omitted.
- Results may be time-dependent: ongoing communication initiatives may change perceptions of appropriate terms for the concept of 'overdiagnosis'.

INTRODUCTION

There is growing concern about the potential for medical tests to detect asymptomatic disease that would never have become clinically apparent or resulted in death (1). 'Overdiagnosis' can harm, primarily via the subsequent risks and costs associated with unnecessary treatment but also via opportunity costs to a healthcare service due to overuse of scarce resources (2).

For peer review only

Various initiatives have aimed to propagate understanding of the concept among patients and the public so that they can make better informed decisions about their health (e.g. 3–5). However, previous studies have found that the concept is challenging to communicate to lay people (e.g. in the context of breast cancer screening; 6,7).

Little is known about how to improve communication of overdiagnosis (8). One barrier is that the term 'overdiagnosis' itself may be confusing (e.g. since it resembles 'misdiagnosis'; 9). Hence, this study asked a large sample of the public in England to appraise descriptions of the concept and tested which of several possible terms people thought made sense as labels (i.e. endorsement). Alternatives included 'overdetection' or 'unnecessary diagnosis' (10) as well as terms related to unnecessary therapy (e.g. 'overtreatment') and terms that were less technically accurate but still had the potential to be seen as applicable (e.g. 'false positives'; 9, 11). A limitation to the utility of 'overdiagnosis' as a label may be the currently low level of public awareness of the term (9, 12). Consequently, this study also measured which terms people had encountered before (i.e. familiarity) to test whether any alternatives were notably more familiar.

Information was derived from materials that have been widely used by the NHS Breast Screening Programme (13) and the Prostate Cancer Risk Management Programme (14). The study was carried out as part of Wave 3 of the Attitudes, Behaviour and Cancer UK Survey (ABACUS). Other studies arising from this survey have also been published (e.g. 15).

METHODS

Design

Institutional approval was granted by the University College London Research Ethics Committee (5771/002). The focus of the present study was on appropriate terminology for

For peer review only

widely used descriptions of overdiagnosis. However, the ABACUS survey was a broader piece of work that also compared the perceived clarity of different forms of overdiagnosis information. Hence, elements of the design and measures have also been reported elsewhere (15). Face-to-face computer-assisted interviews were conducted by TNS (a market research company) as part of a weekly omnibus survey and took place in participants' homes between April and May 2016. Participants were informed:

"We would now like to ask you some questions about leaflets on health-related topics. The NHS offers people a variety of screening tests to check for illnesses before symptoms have appeared. People offered an NHS screening test are often given a leaflet that explains the risks and benefits of having the test. The leaflet is either posted or given out by a doctor or nurse."

Participants then received information on overdiagnosis adapted from written material used by the NHS in England as part of either i) the Breast Screening Programme or ii) the Prostate Cancer Risk Management Programme as they existed in February 2016 (neither of which used a specific label), allocated at random in a 1:1 ratio:

"The test can find an illness that would never have caused a person harm. Some people will be diagnosed and treated for an illness that would never otherwise have been found and would not have become life-threatening." From the breast screening information leaflet (13)

"The test may make you worry by finding an illness that may never cause any symptoms or shorten your life." From the prostate screening information leaflet (14)

References to cancer were removed so that findings could be generalised to beyond these two specific screening contexts.

Participants

For peer review only

Participants were members of the public in England aged 18 to 70 years. National representativeness was sought via a two-stage process. In the first instance, random location sampling was carried out using the Postcode Address File and Census statistics. Within each selected location, participant quotas were set based on demographic characteristics (e.g. age, gender, employment status).

Measures

Demographics: Relevant measures consisted of age, gender, ethnicity, social grade (16), marital status, highest level of education obtained, personal history of cancer, and whether anyone they know had been diagnosed with cancer.

Familiarity with screening information: Participants were asked whether they had ever previously received information about screening (for cancer or other illnesses) via i) reading a leaflet from the NHS, ii) reading an NHS website, and iii) talking with a doctor or nurse (response options: *"yes"*, *"no"*, *"not sure"*).

If participants were eligible for cancer screening and had not been diagnosed with the target disease, they were asked about their previous participation via items designed using the Precaution Adoption Process Model (PAPM; 17), a stage theory of protective health behaviour. This measures whether participants have heard of a particular type of screening, whether they have heard of a type of screening but have never been invited, whether they have been invited but never participated, whether they have participated but not consistently, and whether they have participated consistently. Eligibility criteria and exact question wording for assessing experience have been reported elsewhere (15).

For peer review only

Perceived clarity of information: After reading the information allocated at random, participants were asked, *"How clear do you find this description of a risk of the test?"* with available responses of *"extremely clear"*, *"very clear"*, *"moderately clear"*, *"slightly clear"*, and *"not at all clear"*. This was followed by a question on whether they had read or heard similar information about a screening test before (possible responses were: *"yes"*, *"no"*, and *"not sure"*).

Main outcomes: The first main outcome measure (endorsement) consisted of "Does this term make sense to you as a way of describing this risk of a screening test?", followed by seven terms: "overdiagnosis", "overdetection", "unnecessary diagnosis", "overtreatment", "unnecessary treatment", "false positive diagnosis", and "false positive test results"[1] (responses: "yes", "no"). Suggested terms were based on previous literature (e.g. 10) and group discussion among the research team.

The second outcome measure (familiarity) was: *"Have you ever seen or heard any of the previous seven terms before today?"* and if participants responded *"yes"*, they were asked which out of the previous terms (responses: *"yes"*, *"no"*, *"not sure"*).

Analysis

Participants were excluded if they declined to answer any of the measures included in this study. Methods of coding ethnicity, marital status, highest level of education obtained, social

[1] Participants were also asked, *"Can you think of any other terms to describe this risk that would make sense to you?"*. However, only approximately 60 responses suggesting potentially applicable terms were recorded across all participants (e.g. *"oversensitivity"*, *"unknown diagnosis"*), of which most were mentioned just once and so no further analysis was attempted.

For peer review only

grade, and previous cancer screening participation (i.e. whether they had ever taking part in each form of screening for which they were eligible) have been reported elsewhere (15).

Sample characteristics were summarised with descriptive statistics. We report the percentages of participants (with binomial 95% confidence intervals) who had previously encountered and endorsed each possible number of terms (from 0 to 7 in both cases), and the percentages of participants who had previously encountered and endorsed each specific term.

RESULTS

Participant characteristics

After excluding 223 participants with missing data, 1,888 cases were analysed. Sample characteristics are presented in Table 1. Mean age was 43.6 years (standard deviation: 15.7).

Familiarity with terms

Table 2 illustrates that the majority of participants (58.0%) were not familiar with any of the listed terms. However, the second most common number of terms recognised as familiar was all seven (12.3%). The percentages of participants previously encountering between one and six terms ranged from 1.1% to 9.4%. Table 2 also shows that the percentages of participants who had encountered each specific term ranged from 15.9% (*"overdetection"*) to 28.3% (*"false positive test results"*).

For peer review only

Table 1 – Characteristics of the sample

	Total N=1,888	
**Characteristic**	**n**	**(%)**
**Overdiagnosis information**		
Breast screening text	987	(52.3)
Prostate screening text	901	(47.7)
**Gender**		
Male	879	(46.6)
Female	1,009	(53.4)
**Ethnicity**		
White British	1,432	(75.8)
Other ethnic groups	456	(24.2)
**Marital status**		
Married or living as a couple	1,133	(60.0)
Single, widowed, divorced, or separated	755	(40.0)
**Highest level of education**[1]		
No formal qualifications	281	(14.9)
Approximately Level 1, 2, or 3	890	(47.1)
Approximately Level 4	507	(26.9)
Other	199	(10.5)
Don't know/Not sure	11	(0.6)
**Social class grade**[2]		
Grade A or B	386	(20.4)
Grade C1 or C2	931	(49.3)
Grade D or E	571	(30.2)
**Personal diagnosis of cancer**		
Yes	98	(5.2)
No	1,790	(94.8)
**Knows someone with cancer**		
Yes	1,113	(59.0)
No	771	(40.8)
Don't know/Not sure	4	(0.2)
**Any previous cervical screening experience**		
Yes	652	(34.5)
No	226	(12.0)
Not eligible	1,010	(53.5)
**Any previous breast screening experience**		
Yes	317	(16.8)
No	83	(4.4)
Not eligible	1,488	(78.8)
**Any previous bowel screening experience**		
Yes	293	(15.5)
No	181	(9.6)
Not eligible	1,414	(74.9)
**Any previous prostate screening experience**		
Yes	77	(4.1)
No	344	(18.2)
Not eligible	1,467	(77.7)
**Previously read a screening leaflet**		
Yes	1,058	(56.0)
No	809	(42.8)
Don't know/Not sure	21	(1.1)
**Previously read an NHS screening website**		
Yes	324	(17.2)
No	1,549	(82.0)
Don't know/Not sure	15	(0.8)
**Discussed screening with doctor/nurse**		
Yes	624	(33.1)
No	1,251	(66.3)
Don't know/Not sure	13	(0.7)

For peer review only

**Previously read or heard similar information**		
Yes	662	(35.1)
No	1,185	(62.8)
Don't know/Not sure	41	(2.2)
**Perceived clarity of information**		
Extremely clear	170	(9.0)
Very clear	636	(33.7)
Moderately clear	685	(36.3)
Slightly clear	206	(10.9)
Not at all clear	191	(10.1)

[1]Level 1-3 qualifications include e.g. GCSEs and A Levels; Level 4 qualifications include degrees and higher degrees
[2]Social grades are based on occupation (e.g. Grade A includes managerial roles; Grade E includes casual workers)

For peer review only

Table 2 – Number of participants (and percentages and 95% CIs) previously encountering i) each number of possible terms and ii) each specific term. Participants receiving overdiagnosis information based on the Breast Screening programme or the Prostate Cancer Risk Management Programme were analysed separately.

		Number of terms previously encountered: n, %, 95% CIs							
**Screening information**	Total	0	1	2	3	4	5	6	7
Breast	n (%)	583 (59.1)	73 (7.4)	76 (7.7)	53 (5.4)	41 (4.2)	28 (2.8)	9 (0.9)	124 (12.6)
N=987	95% CI	56.0 to 62.1	5.9 to 9.2	6.2 to 9.5	4.1 to 6.9	3.0 to 5.5	1.9 to 4.0	0.5 to 1.7	10.6 to 14.7
Prostate	n (%)	512 (56.8)	64 (7.1)	101 (11.2)	47 (5.2)	37 (4.1)	19 (2.1)	12 (1.3)	109 (12.1)
n=901	95% CI	53.6 to 60.0	5.6 to 8.9	9.3 to 13.4	3.9 to 6.8	3.0 to 5.6	1.3 to 3.2	0.7 to 2.2	10.1 to 14.3
Total	n (%)	1,095 (58.0)	137 (7.3)	177 (9.4)	100 (5.3)	78 (4.1)	47 (2.5)	21 (1.1)	233 (12.3)
N=1,888	95% CI	55.8 to 60.2	6.2 to 8.5	8.1 to 10.8	4.4 to 6.4	3.3 to 4.1	1.9 to 3.3	0.7 to 1.7	10.9 to 13.9
		"Which of the following term or terms had you heard of before?": n, %, 95% CIs							
**Screening information**	Total	Not sure	Overdiagnosis	Overdetection	Unnecessary diagnosis	Overtreatment	Unnecessary treatment	False positive diagnosis	False positive test result
Breast	n (%)	7 (0.7)	215 (21.8)	163 (16.5)	214 (21.7)	225 (22.8)	280 (28.4)	243 (24.6)	270 (27.4)
N=987	95% CI	0.3 to 1.4	19.3 to 24.4	14.3 to 18.9	19.2 to 24.3	20.3 to 25.5	25.6 to 31.2	22.0 to 27.4	24.6 to 30.2
Prostate	n (%)	14 (1.6)	196 (21.8)	137 (15.2)	216 (24.0)	186 (20.6)	253 (28.1)	232 (25.7)	265 (29.4)
n=901	95% CI	0.9 to 2.5	19.2 to 24.5	13.0 to 17.7	21.3 to 26.8	18.1 to 23.4	25.2 to 31.1	23.0 to 28.7	26.5 to 32.4
Total	n (%)	21 (1.1)	411 (21.8)	300 (15.9)	430 (22.8)	411 (21.8)	533 (28.2)	475 (25.2)	535 (28.3)
N=1,888	95% CI	0.7 to 1.7	20.0 to 23.7	14.3 to 17.6	20.9 to 24.7	20.0 to 23.7	26.2 to 30.3	23.2 to 27.2	26.3 to 30.4

For peer review only

Table 3 – Number of participants (and percentages and 95% CIs) endorsing i) each number of possible terms and ii) each specific term as making sense to them

		Number of terms endorsed as making sense as a way of describing the text: n, %, 95% CIs							
**Screening information**	Total	0	1	2	3	4	5	6	7
Breast	n (%)	429 (43.5)	76 (7.7)	86 (8.7)	74 (7.5)	67 (6.8)	58 (5.9)	42 (4.3)	155 (15.7)
n=987	95% CI	40.4 to 46.6	6.2 to 9.5	7.1 to 10.6	6.0 to 9.3	5.3 to 8.5	4.5 to 7.5	3.1 to 5.7	13.5 to 18.1
Prostate	n (%)	408 (45.3)	59 (6.5)	73 (8.1)	68 (7.5)	66 (7.3)	48 (5.3)	37 (4.1)	142 (15.8)
n=901	95% CI	42.1 to 48.5	5.1 to 8.3	6.5 to 10.0	6.0 to 9.4	5.8 to 9.2	4.0 to 6.9	3.0 to 5.6	13.5 to 18.2
Total	n (%)	837 (44.3)	135 (7.2)	159 (8.4)	142 (7.5)	133 (7.0)	106 (5.6)	79 (4.2)	297 (15.7)
N=1,888	95% CI	42.1 to 46.6	6.1 to 8.4	7.2 to 9.7	6.4 to 8.8	6.0 to 8.3	4.6 to 6.7	3.4 to 5.2	14.1 to 17.4

		"Which terms make sense as a way of describing this risk of a screening test?": n, %, 95% CIs						
**Screening information**	Total	Overdiagnosis	Overdetection	Unnecessary diagnosis	Overtreatment	Unnecessary treatment	False positive diagnosis	False positive test results
Breast	n (%)	334 (33.8)	275 (27.9)	349 (35.4)	336 (34.0)	409 (41.4)	324 (32.8)	338 (34.2)
n=987	95% CI	30.9 to 36.8	25.1 to 30.7	32.4 to 38.4	31.1 to 37.0	38.4 to 44.5	30.0 to 35.8	31.3 to 37.2
Prostate	n (%)	291 (32.3)	247 (27.4)	307 (34.1)	280 (31.1)	354 (39.3)	320 (35.5)	330 (36.6)
n=901	95% CI	29.3 to 35.4	24.6 to 30.4	31.0 to 37.2	28.1 to 34.2	36.1 to 42.5	32.4 to 38.7	33.5 to 39.8
Total	n (%)	625 (33.1)	522 (27.6)	656 (34.7)	616 (32.6)	763 (40.4)	644 (34.1)	668 (35.4)
N=1,888	95% CI	31.0 to 35.3	25.7 to 29.7	32.6 to 36.9	30.5 to 34.8	38.2 to 42.6	32.0 to 36.3	33.2 to 37.6

For peer review only

Terms endorsed as making sense

Table 3 shows that participants most commonly did not endorse any terms as making sense (44.3%). As with results for familiarity, the second most common number of terms endorsed was all seven (15.7%). The most commonly endorsed term was *"unnecessary treatment"* (40.4% of participants in the sample endorsed this term overall; 41.4% and 39.3% after being given the breast and prostate information, respectively). Endorsement of other terms ranged from 27.9% (*"overdetection"*) to 35.4% (*"unnecessary diagnosis"*) for information from breast screening and 27.4% (*"overdetection"*) to 36.6% (*"false positive test results"*) for information from prostate screening.

DISCUSSION

This study aimed to address a gap in the literature regarding how best to communicate the concept of overdiagnosis (8). We investigated whether people are familiar with terms used to describe overdiagnosis or related concepts, and whether these made sense as labels for widely used descriptions. We found that a majority of participants (58.0%) had never encountered any of the assessed terms before and a large proportion (44.3%) did not rate any term as making sense as a suitable label for either of two widely used descriptions of the concept. In addition, no specific term(s) emerged as being notably more familiar or applicable.

A previous similar study found that only 30.0% of the UK general public (aged 50-70) had previously encountered the specific term, 'overdiagnosis' (9), which is broadly comparable to the 21.8% who reported having encountered it in this study. Our findings are also consistent with those of a previous study that was part of redesign of the information materials for the NHS Breast Screening Programme (18). Women were interviewed with the aim of finding ways to

communicate the concept of overdiagnosis: participants struggled to understand it, regardless of whether it was labelled as ‘overdiagnosis’ or ‘overtreatment’.

Terminology can have a role in transferring information to lay individuals as part of a description of technical concepts (10,19). However, the main implication of our results is that currently there may only be limited value in using any of the labels that we tested. Terms used were neither familiar nor rated as particularly relevant in this large, diverse sample, meaning that they may be confusing, unintuitive, and hard for people to memorise and recall. Even the most technically appropriate terms (e.g. ‘overdiagnosis’ and ‘overdetection’) were not endorsed as making sense more frequently than suggested alternative terminology (e.g. ‘unnecessary diagnosis) or more inaccurate and potentially problematic terminology (e.g. ‘false positive diagnosis’). To some extent, the limited familiarity and low levels of endorsement at present supports UK policy decisions not to use a specific term in the revised breast screening information leaflet (18). Furthermore, shortly before the start of recruitment, an updated NHS information leaflet regarding PSA screening was published, with additional detail on overdiagnosis and overtreatment (20); this also omitted any specific descriptive label.

One unexpected finding was that the second most common number of terms that were familiar or endorsed was all seven (12.3% and 15.7%, respectively). This may be a reflection of differences between terms being subtle: members of the public may not recall exactly which terms they had encountered but felt that broader concepts like diagnosis and treatment were familiar.

Continuing communication efforts mean that these results may change over time: it is plausible that ongoing campaigns (3-5) will result in the public becoming increasingly familiar with the concept and terminology, having a better understanding of the differences between similar

For peer review only

terms (e.g. seeing ‘false positives’ as distinct from ‘overdiagnosis’), and being more likely to endorse particular terms as applicable. However, present attempts to communicate overdiagnosis (e.g. as part of screening invitations or mainstream media stories) may be more effective if explicit descriptions are used, such as adapted forms of information from the Breast Screening Programme. This has undergone an extensive design process (18) and is more likely to be rated as clearer than the equivalent from the Prostate Cancer Risk Management Programme. However, fewer than half of participants (46.6%) rated it as very or extremely clear, suggesting that future research could be used to improve it further (e.g. by providing additional detail; 15).

This study has limitations. The list of terms tested was not exhaustive; there may be suitable terminology that we did not assess. However, we found very similar levels of endorsement for terms that used several variations on the themes of diagnosis, treatment, and false positives, providing some evidence that any superior terms would have to be quite different. In addition, as noted in our previous study, descriptions of overdiagnosis were brief and presented outside the context of the original written information about screening from which they were adapted; comprehension may be different (either better or worse) in the presence of additional detail (15); this warrants further research.

Conclusions

It has been suggested that alternative terminology to ‘overdiagnosis’ would help increase awareness and understanding of the concept among individuals who may face healthcare decisions that would put them at risk. These results suggest that, at present, using specific terms would have limited benefits. It may currently be more effective to refer to the concept via more explicit descriptions. Previous research indicates that information from the NHS Breast

For peer review only

Screening Programme may be a relatively effective template for this purpose. However, future research could explore scope for improvement.

Contributors: AG, CR, and JW conceived and designed the study. AG analysed the data. AG, CR, and JW participated in the interpretation of results. AG, CR, and JW drafted the manuscript, participated in critical revision, and approved the final version.

Funding: This work was supported by a programme grant from Cancer Research UK awarded to Prof Jane Wardle [C1418/A14134]. Dr Jo Waller is supported by a Career Development Fellowship from Cancer Research UK [C7492/A17219]. Cancer Research UK was not involved in the design of this study; the collection, analysis, or interpretation of the results; in the writing of the manuscript; or in the decision to submit for publication.

Competing interests: None declared.

Patient consent: Obtained.

Data sharing: No additional data are available.

REFERENCES

1. Heath I. Overdiagnosis: when good intentions meet vested interests — an essay by Iona Heath. Br Med J. 2013;347:f6361.

2. Moynihan R, Doust J, Henry D. Preventing overdiagnosis: how to stop harming he healthy. Br Med J. 2012;344:e3502.

3. Glasziou P, Moynihan R, Richards T. Godlee F. Too much medicine; too little care. Br Med J. 2013;347:f4247.

For peer review only

4. Malhotra A, Maughan D, Ansell J, et al. Choosing Wisely in the UK: the Academy of Medical Royal Colleges' initiative to reduce the harms of too much medicine. Br Med J. 2015;350:h2308.

5. Calderwood C. Chief Medical Officer's annual report 2014-15. 2016. http://www.gov.scot/Publications/2016/01/3745/0

6. Hersch J, Jansen J, Barratt A, et al. Women's views on overdiagnosis in breast cancer screening: a qualitative study. Br Med J. 2013;346:f158.

7. Waller J, Douglas E, Whitaker KL, Wardle J. Women's responses to information about overdiagnosis in the UK breast cancer screening programme: a qualitative study. BMJ Open. 2013;3:e002703.

8. McCaffery KJ, Jansen J, Scherer LD, et al. Walking the tightrope: communicating overdiagnosis in modern healthcare. Br Med J. 2016;352:i348.

9. Ghanouni A, Meisel SF, Renzi C, Wardle J, Waller J. Public definitions of the term 'overdiagnosis' in the UK. BMJ Open. 2016;:e010723.

10. Hersch J, Jansen J, Barratt A, et al. Overdetection in breast cancer screening: development and preliminary evaluation of a decision aid. BMJ Open. 2014;4:e006016.

11. Moynihan R, Nickel B, Hersch J, et al. What do you think overdiagnosis means? A qualitative analysis of responses from a national community survey of Australians. BMJ Open. 2015;5:e007436.

12. Moynihan R, Nickel B, Hersch J, et al. Public opinions about overdiagnosis: a national community survey. PLoS One. 2015;10:e0125165.

For peer review only

13. NHS Breast Screening Programme. NHS Breast Screening: Helping you decide. 2013. Available from: https://www.gov.uk/government/uploads/system/uploads/attachment_data/file/440798/nhsbsp.pdf

14. Burford D, Austoker J, Kirby M. PSA (prostate specific antigen) testing for prostate cancer. An information sheet for men considering a PSA test. 2009.

15. Ghanouni A, Renzi C, McBride E, Waller J. Comparing perceived clarity of information on overdiagnosis used for breast and prostate cancer screening in England: an experimental survey. BMJ Open. 2017;7:e015955.

16. National Readership Survey. Social grade. http://www.nrs.co.uk/nrs-print/lifestyle-and-classification-data/social-grade/ Accessed: 1st December 2017.

17. Weinstein ND. The precaution adoption process. Heal Psychol. 1988;7:355–86.

18. Forbes LJ, Ramirez AJ, Expert group on Information about Breast Screening. Offering informed choice about breast screening. J Med Screen. 2014;21:194–200.

19. Fage-Butler AM, Nisbeth Jensen M. Medical terminology in online patient-patient communication: evidence of high health literacy? Heal Expect. 2016;19:643–53.

20. Public Health England. PSA testing and prostate cancer: advice for well men aged 50 and over. 2016. Available from: https://www.gov.uk/government/uploads/system/uploads/attachment_data/file/509191/Patient_info_sheet.pdf

For peer review only

BMJ Open

Improving public understanding of 'overdiagnosis' in England: a population survey assessing familiarity with possible terms for labelling the concept and perceptions of appropriate terminology

Journal:	*BMJ Open*
Manuscript ID	bmjopen-2017-021260.R1
Article Type:	Research
Date Submitted by the Author:	02-Mar-2018
Complete List of Authors:	Ghanouni, Alex; University College London, Research Department of Behavioural Science and Health<br>Renzi, Cristina; UCL, Research Department of Behavioural Science and Health<br>Waller, Jo; UCL, Research Department of Behavioural Science and Health
**Primary Subject Heading**:	Communication
Secondary Subject Heading:	Epidemiology, Public health, Health services research
Keywords:	EPIDEMIOLOGY, PUBLIC HEALTH, Health policy < HEALTH SERVICES ADMINISTRATION & MANAGEMENT

SCHOLARONE™ Manuscripts

For peer review only

FULL TITLE PAGE

Title: Improving public understanding of 'overdiagnosis' in England: a population survey assessing familiarity with possible terms for labelling the concept and perceptions of appropriate terminology

Manuscript type: Research article

Authors:

Alex Ghanouni[a] a.ghanouni@ucl.ac.uk

Cristina Renzi[a] c.renzi@ucl.ac.uk

Jo Waller[ab] j.waller@ucl.ac.uk | Tel: +44 (0)20 7679 5958 | Fax: +44 (0)20 7679 8354

Affiliations:

[a]Research Department of Behavioural Science and Health, University College London, Gower Street, London, WC1E 6BT, United Kingdom

[b]Corresponding author

Word count: Abstract: 299/300; Body: 3,332/4,000; References: 23; Tables or figures: 5

For peer review only

ABSTRACT

Objectives: Communicating the concept of 'overdiagnosis' to lay individuals is challenging, partly because the term itself is confusing. This study tested whether alternative descriptive labels may be more appropriate.

Design: Questionnaire preceded by a description of overdiagnosis.

Setting: Home-based, computer-assisted face-to-face survey.

Participants: 2,111 adults aged 18 to 70 years in England recruited using random location sampling by a survey company. Data from 1,888 participants were analysed after exclusions due to missing data.

Interventions: Participants were given one of two pieces of text describing overdiagnosis, allocated at random, adapted from NHS breast and prostate cancer screening leaflets.

Primary and secondary outcome measures: Main outcomes were which of several available terms (e.g. 'overdetection') participants had previously encountered and which they endorsed as applicable labels for the concept described. Demographics and previous exposure to screening information were also measured. Main outcomes were summarised with descriptive statistics. Predictors of previously encountering at least one term, or endorsing at least one as making sense, were assessed using binary logistic regression.

Results: 58.0% of participants had not encountered any suggested term; 44.0% did not endorse any as applicable labels. No term was notably familiar; the proportion of participants who had previously encountered each term ranged from 15.9 to 28.3%. Each term was only endorsed as applicable by a minority (range: 27.6% to 40.4%). Notable predictors of familiarity included

For peer review only - http://bmjopen.bmj.com/site/about/guidelines.xhtml

education, age, and ethnicity; participants were less likely to have encountered terms if they were older, not White British or had less education, Findings were similar for both pieces of information.

Conclusions: Familiarity with suggested terms for overdiagnosis and levels of endorsement were low and no clear alternative labels for the concept were identified, suggesting that changing terminology alone would do little to improve understanding, particularly for some population groups. Explicit descriptions may be more effective.

Keywords: Decision Making; Surveys and Questionnaires; Early Detection of Cancer; Mass Screening; Medical Overuse

Strengths and limitations of this study

- This study assessed familiarity and perceived appropriateness of a broad range of possible terms for the concept of 'overdiagnosis' among a large, representative sample of the general public in England.
- The concept was described based on information that was widely used by the National Health Service in England, maintaining generalisability.
- The list of possible terms was not exhaustive; more suitable alternative labels may have been omitted.
- Results may be time-dependent: ongoing communication initiatives may change perceptions of appropriate terms for the concept of 'overdiagnosis'.

INTRODUCTION

For peer review only

There is growing concern among healthcare providers and policymakers about the potential for medical tests to detect asymptomatic disease that would never have become clinically apparent or resulted in death (1). ‘Overdiagnosis’ can harm, primarily via the subsequent risks and costs associated with unnecessary treatment but also via opportunity costs to a healthcare service due to overuse of scarce resources (2). Various initiatives have aimed to propagate understanding of the concept among patients and the public so that they can make better informed decisions about their health (e.g. 3–5). However, previous studies have found that the concept is challenging to communicate to lay people (e.g. in the context of breast cancer screening; 6,7).

Little is known about how to improve communication of overdiagnosis (8). One barrier is that the term ‘overdiagnosis’ itself may be confusing and counterintuitive. For example, a previous study in the UK asked members of the public to give their interpretation of what the word meant; a large proportion of participants gave definitions that did not match the concept but resembled other similar words (e.g. ‘providing an incorrect diagnosis’ since 'overdiagnosis' resembles 'misdiagnosis; 9). Equivalent findings have been reported by a previous survey in Australia (10). In addition, a focus group study undertaken as part of the development of a decision aid for breast screening found that 'overdiagnosis' was not understood intuitively and that ‘overdetection’ may be clearer (11).

These findings suggest that the term ‘overdiagnosis’ does not clearly reflect its intended meaning for the general public and that other terms (such as 'overdetection') may be more appropriate. Hence, this study asked a large sample of the public in England to appraise descriptions of the concept and tested which of several possible terms people thought made sense as labels (i.e. endorsement). Alternatives included ‘overdetection’ or ‘unnecessary

For peer review only - http://bmjopen.bmj.com/site/about/guidelines.xhtml

diagnosis' (11) as well as terms related to unnecessary therapy (e.g. 'overtreatment') and terms that were less technically accurate but still had the potential to be seen as applicable (e.g. 'false positives'; 9, 10). A limitation to the utility of 'overdiagnosis' as a label may be the currently low level of public awareness of the term (9, 12). Consequently, this study also measured which terms people had encountered before (i.e. familiarity) to test whether any alternatives were notably more familiar. We also explored whether participant characteristics were associated with having previously encountered these terms, or with endorsing them as making sense, as this may aid targeted communication efforts.

Information was derived from materials that have been widely used by the NHS Breast Screening Programme (13) and the Prostate Cancer Risk Management Programme (14). The study was carried out as part of Wave 3 of the Attitudes, Behaviour and Cancer UK Survey (ABACUS). Other studies arising from this survey have also been published (e.g. 15).

METHODS

Design

Institutional approval was granted by the University College London Research Ethics Committee (5771/002). The focus of the present study was on appropriate terminology for widely used descriptions of overdiagnosis. However, the ABACUS survey was a broader piece of work that also compared the perceived clarity of different forms of overdiagnosis information. Hence, elements of the design and measures have also been reported elsewhere (15). Face-to-face computer-assisted interviews were conducted by TNS (a market research company) as part of a weekly omnibus survey and took place in participants' homes between April and May 2016. Participants were informed:

For peer review only

"We would now like to ask you some questions about leaflets on health-related topics. The NHS offers people a variety of screening tests to check for illnesses before symptoms have appeared. People offered an NHS screening test are often given a leaflet that explains the risks and benefits of having the test. The leaflet is either posted or given out by a doctor or nurse."

Participants then received information on overdiagnosis adapted from written material used by the NHS in England as part of either i) the Breast Screening Programme or ii) the Prostate Cancer Risk Management Programme as they existed in February 2016 (neither of which used a specific label), allocated at random in a 1:1 ratio:

"The test can find an illness that would never have caused a person harm. Some people will be diagnosed and treated for an illness that would never otherwise have been found and would not have become life-threatening." From the breast screening information leaflet (13)

"The test may make you worry by finding an illness that may never cause any symptoms or shorten your life." From the prostate screening information leaflet (14)

The Breast Screening Programme in England offers triennial mammography to all women between the ages of 50 to 70 years and registered with a General Practitioner. The Prostate Screening Risk Management Programme allows men aged 50 years or older to have a Prostate Specific Antigen test by requesting it from their GP after discussing the risks and benefits. References to cancer were removed so that findings could be generalised to beyond these two specific screening contexts.

Participants

Participants were members of the public in England aged 18 to 70 years. National representativeness was sought via a two-stage process. In the first instance, random location

For peer review only

sampling was carried out using the Postcode Address File and Census statistics. Within each selected location, participant quotas were set based on demographic characteristics (i.e. age, gender, employment status, and presence of children in the home).

Measures

Demographics: Relevant measures consisted of age, gender, ethnicity, social grade (16), marital status, highest level of education obtained, personal history of cancer (*"Have you ever been diagnosed with cancer?"*; *"yes"* or *"no"*), and whether anyone they know had been diagnosed with cancer (*"Has anyone close to you ever been diagnosed with cancer?"*; *"yes"*, *"no"*, or *"don't know"*).

Familiarity with screening information: Participants were asked whether they had ever previously received information about screening (for cancer or other illnesses) via i) reading a leaflet from the NHS, ii) reading an NHS website, and iii) talking with a doctor or nurse (response options: *"yes"*, *"no"*, *"not sure"*).

If participants were eligible for cancer screening and had not been diagnosed with the target disease, they were asked about their previous participation (e.g. only women aged 47-70 years without a breast cancer diagnosis were asked about breast screening participation) via items designed using the Precaution Adoption Process Model (PAPM; 17), a stage theory of protective health behaviour. This measures whether participants have heard of a particular type of screening, whether they have heard of a type of screening but have never been invited, whether they have been invited but never participated, whether they have participated but not consistently, and whether they have participated consistently. Eligibility criteria and exact question wording for assessing experience have been reported elsewhere (15).

Perceived clarity of information: After reading the information allocated at random, participants were asked, *"How clear do you find this description of a risk of the test?"* with available responses of *"extremely clear"*, *"very clear"*, *"moderately clear"*, *"slightly clear"*, and *"not at all clear"*. This was followed by a question on whether they had read or heard similar information about a screening test before (possible responses were: *"yes"*, *"no"*, and *"not sure"*).

Main outcomes: The first main outcome measure (endorsement) consisted of *"Does this term make sense to you as a way of describing this risk of a screening test?"*, followed by seven terms: *"overdiagnosis"*, *"overdetection"*, *"unnecessary diagnosis"*, *"overtreatment"*, *"unnecessary treatment"*, *"false positive diagnosis"*, and *"false positive test results"*[1] (responses: *"yes"*, *"no"*). Suggested terms were based on previous literature (e.g. 11) and discussion among the research team.

The second outcome measure (familiarity) was: "Have you ever seen or heard any of the previous seven terms before today?" and if participants responded *"yes"*, they were asked which out of the previous terms (responses: *"yes"*, *"no"*, *"not sure"*).

Ancillary measures: The measures used in this study were part of the broader ABACUS survey. Most of the items in this study were presented at the start of the survey and so the effects of priming from unrelated items were expected to be minimal. In order, the full survey included: questions on decision-making style and previous exposure to cancer screening information,

[1] Participants were also asked, *"Can you think of any other terms to describe this risk that would make sense to you?"*. However, only approximately 60 responses suggesting potentially applicable terms were recorded across all participants (e.g. *"oversensitivity"*, *"unknown diagnosis"*), of which most were mentioned just once and so no further analysis was attempted.

For peer review only

information on overdiagnosis adapted from screening leaflets, questions on perceived clarity of this information, previous exposure to similar information, the main study outcomes, and help-seeking behaviour in relation to cancer screening, followed by more questions on self-rated health, questions on cancer diagnoses, perceived cancer risk, screening behaviour in relation to each of the applicable programmes (cervical, breast, bowel, prostate), and educational level.

Public Involvement

Public involvement consisted of input into the design of the study and development of measures via pilot testing of the survey. This comprised two stages: first, a series of telephone cognitive interviews (18) was used to determine whether items could be understood by lay people (n=11) and that the survey was not overly burdensome. Participants were asked for feedback on any items they found difficult to understand or answer and the survey was revised accordingly. Second, a web-based version of the survey was pilot-tested with 431 participants.

There are currently no plans to disseminate the results to study participants.

Analysis

Participants were excluded if they declined to answer any of the measures included in this study. Ethnicity and marital status were dichotomised into “White British” and “other ethnic groups”, and “single, widowed, divorced or separated” and “married or living as a couple”, respectively. Social class grade was categorised as “grades A or B”, “grades C1 or C2”, and “grades D or E”. Education was coded based on Levels 1-4 from the Office of National Statistics (19) or “Other” for non-ordinal levels of education such as professional qualifications.

Sample characteristics were summarised with descriptive statistics. We report the percentages of participants (with binomial 95% confidence intervals) who had i) previously encountered and

For peer review only

ii) endorsed each possible number of terms (from 0 to 7 in both cases), and the percentages of participants who had previously encountered and endorsed each specific term.

Responses to i) and ii) were also recoded into “previously seen or heard one or more terms” (familiarity) and “endorsed one or more terms as making sense to them” (endorsement) for further evaluation e.g. “was familiar with at least one term” vs. “was not familiar” (including responses of “not sure”). Two exploratory logistic regression models tested the null hypothesis that predictor variables were unrelated to either of these outcomes. In both models, predictor variables consisted of demographic characteristics, familiarity with screening information, the overdiagnosis information condition (breast vs. prostate), and perceived clarity. In addition, each model included the outcome from the other as a predictor (e.g. the endorsement model included familiarity as a potential predictor variable). Variance Inflation Factors were small (all <2.969 and <2.972, respectively), indicating that (multi)collinearity was limited and a Box-Tidwell procedure found little evidence to suggest that the age variable violated the assumption of linearity (p-values: .110 and .508). Adjusted odds-ratios (ORs), accompanying 95% CIs, and p-values for having previously seen or heard one or more terms before and endorsing one or more term as making sense are reported alongside descriptive statistics.

RESULTS

Participant characteristics

After excluding 223 participants with missing data, 1,888 cases were analysed. Sample characteristics are presented in Table 1. Mean age was 43.6 years (standard deviation: 15.7).

Familiarity with terms

For peer review only

Table 2 illustrates that the majority of participants (58.0%) were not familiar with any of the listed terms. However, the second most common number of terms recognised as familiar was all seven (12.3%). The percentages of participants previously encountering between one and six terms ranged from 1.1% to 9.4%. Table 2 also shows that the percentages of participants who had encountered each specific term ranged from 15.9% (*"overdetection"*) to 28.3% (*"false positive test results"*).

Table 3 reports predictors of familiarity with at least one term. Among demographic characteristics, there was strong evidence against the null hypothesis for ethnicity, education, and age: White British participants were more likely to be familiar with at least one term compared with other ethnic groups. Participants with a higher (or 'other') level of education were also more likely to be familiar with one or more terms compared with participants who had no formal qualifications (or did not know). Older participants were less likely to be familiar with one or more terms. Participants were also more likely to be familiar with one or more terms if they knew someone with cancer, had previously read an NHS website about screening, discussed screening with a doctor or nurse, had previously read or seen similar information, or endorsed one or more terms as making sense as labels.

Finally, there was moderate evidence against the null hypothesis for an association with social class grade with participants in grades A or B being more likely to be familiar with at least one term compared with participants in grades D or E.

Terms endorsed as making sense

Table 4 shows that participants most commonly did not endorse any terms as making sense (44.3%). As with results for familiarity, the second most common number of terms endorsed was all seven (15.7%). The most commonly endorsed term was *"unnecessary treatment"* (40.4% of

For peer review only

participants in the sample endorsed this term overall; 41.4% and 39.3% after being given the breast and prostate information, respectively). Endorsement of other terms ranged from 27.9% (*"overdetection"*) to 35.4% (*"unnecessary diagnosis"*) for information from breast screening and 27.4% (*"overdetection"*) to 36.6% (*"false positive test results"*) for information from prostate screening.

Other than familiarity (Table 3), the logistic regression analysis of predictors of endorsement only showed strong evidence against the null hypothesis for gender and knowing someone with cancer: males and participants who knew someone diagnosed with cancer were more likely to endorse at least one term as making sense (Table 5).

For peer review only

Table 1 – Characteristics of the sample

	Total N=1,888	
**Characteristic**	**n**	**(%)**
**Overdiagnosis information**		
Breast screening text	987	(52.3)
Prostate screening text	901	(47.7)
**Gender**		
Male	879	(46.6)
Female	1,009	(53.4)
**Ethnicity**		
White British	1,432	(75.8)
Other ethnic groups	456	(24.2)
**Marital status**		
Married or living as a couple	1,133	(60.0)
Single, widowed, divorced, or separated	755	(40.0)
**Highest level of education**[1]		
No formal qualifications	281	(14.9)
Approximately Level 1, 2, or 3	890	(47.1)
Approximately Level 4	507	(26.9)
Other	199	(10.5)
Don't know/Not sure	11	(0.6)
**Social class grade**[2]		
Grade A or B	386	(20.4)
Grade C1 or C2	931	(49.3)
Grade D or E	571	(30.2)
**Personal diagnosis of cancer**		
Yes	98	(5.2)
No	1,790	(94.8)
**Knows someone with cancer**		
Yes	1,113	(59.0)
No	771	(40.8)
Don't know/Not sure	4	(0.2)
**Any previous cervical screening experience**		
Yes	652	(34.5)
No	226	(12.0)
Not eligible	1,010	(53.5)
**Any previous breast screening experience**		
Yes	317	(16.8)
No	83	(4.4)
Not eligible	1,488	(78.8)
**Any previous bowel screening experience**		
Yes	293	(15.5)
No	181	(9.6)
Not eligible	1,414	(74.9)
**Any previous prostate screening experience**		
Yes	77	(4.1)
No	344	(18.2)
Not eligible	1,467	(77.7)
**Previously read a screening leaflet**		
Yes	1,058	(56.0)
No	809	(42.8)
Don't know/Not sure	21	(1.1)
**Previously read an NHS screening website**		
Yes	324	(17.2)
No	1,549	(82.0)
Don't know/Not sure	15	(0.8)
**Discussed screening with doctor/nurse**		
Yes	624	(33.1)
No	1,251	(66.3)
Don't know/Not sure	13	(0.7)
**Previously read or heard similar information**		
Yes	662	(35.1)

For peer review only

No	1,185	(62.8)
Don't know/Not sure	41	(2.2)
**Perceived clarity of information**		
Extremely clear	170	(9.0)
Very clear	636	(33.7)
Moderately clear	685	(36.3)
Slightly clear	206	(10.9)
Not at all clear	191	(10.1)

[1]Level 1-3 qualifications include e.g. GCSEs and A Levels; Level 4 qualifications include degrees and higher degrees

[2]Social grades are based on occupation (e.g. Grade A includes managerial roles; Grade E includes casual workers)

For peer review only

Table 2 – Number of participants (and percentages and 95% CIs) previously encountering i) each number of possible terms and ii) each specific term. Participants receiving overdiagnosis information based on the Breast Screening programme or the Prostate Cancer Risk Management Programme were analysed separately.

		Number of terms previously encountered: n, %, 95% CIs							
**Screening information**	Total	0	1	2	3	4	5	6	7
Breast	n (%)	583 (59.1)	73 (7.4)	76 (7.7)	53 (5.4)	41 (4.2)	28 (2.8)	9 (0.9)	124 (12.6)
N=987	95% CI	56.0 to 62.1	5.9 to 9.2	6.2 to 9.5	4.1 to 6.9	3.0 to 5.5	1.9 to 4.0	0.5 to 1.7	10.6 to 14.7
Prostate	n (%)	512 (56.8)	64 (7.1)	101 (11.2)	47 (5.2)	37 (4.1)	19 (2.1)	12 (1.3)	109 (12.1)
n=901	95% CI	53.6 to 60.0	5.6 to 8.9	9.3 to 13.4	3.9 to 6.8	3.0 to 5.6	1.3 to 3.2	0.7 to 2.2	10.1 to 14.3
Total	n (%)	1,095 (58.0)	137 (7.3)	177 (9.4)	100 (5.3)	78 (4.1)	47 (2.5)	21 (1.1)	233 (12.3)
N=1,888	95% CI	55.8 to 60.2	6.2 to 8.5	8.1 to 10.8	4.4 to 6.4	3.3 to 4.1	1.9 to 3.3	0.7 to 1.7	10.9 to 13.9
		"Which of the following term or terms had you heard of before?": n, %, 95% CIs							
**Screening information**	Total	Not sure	Overdiagnosis	Overdetection	Unnecessary diagnosis	Overtreatment	Unnecessary treatment	False positive diagnosis	False positive test result
Breast	n (%)	7 (0.7)	215 (21.8)	163 (16.5)	214 (21.7)	225 (22.8)	280 (28.4)	243 (24.6)	270 (27.4)
N=987	95% CI	0.3 to 1.4	19.3 to 24.4	14.3 to 18.9	19.2 to 24.3	20.3 to 25.5	25.6 to 31.2	22.0 to 27.4	24.6 to 30.2
Prostate	n (%)	14 (1.6)	196 (21.8)	137 (15.2)	216 (24.0)	186 (20.6)	253 (28.1)	232 (25.7)	265 (29.4)
n=901	95% CI	0.9 to 2.5	19.2 to 24.5	13.0 to 17.7	21.3 to 26.8	18.1 to 23.4	25.2 to 31.1	23.0 to 28.7	26.5 to 32.4
Total	n (%)	21 (1.1)	411 (21.8)	300 (15.9)	430 (22.8)	411 (21.8)	533 (28.2)	475 (25.2)	535 (28.3)
N=1,888	95% CI	0.7 to 1.7	20.0 to 23.7	14.3 to 17.6	20.9 to 24.7	20.0 to 23.7	26.2 to 30.3	23.2 to 27.2	26.3 to 30.4

For peer review only

Table 3 – Familiarity with one or more vs. no terms: descriptive statistics, adjusted ORs, 95% CIs, and p-values for variables in the multivariable binary logistic regression model

		Familiarity with no terms vs. at least one term: n (%)		Adjusted OR, 95% CI	p-value
**Characteristic**	**Total (N=1,888)**	**No term (n=1,095; 58.0%)**	**At least one term (n=793; 42.0%)**	**At least one familiar term (vs. None)**	
**Overdiagnosis information**					
Breast screening text	987	583 (59.1)	404 (40.9)	0.87, 0.70 to 1.09	.224
vs. Prostate screening text	901	512 (56.8)	389 (43.2)		
**Gender**					
Male	879	534 (60.8)	345 (39.2)	1.00, 0.79 to 1.26	.998
vs. Female	1,009	561 (55.6)	448 (44.4)		
**Ethnicity**					
White British	1,432	775 (54.1)	657 (45.9)	2.14, 1.61 to 2.84	**<.0005**
vs. Other ethnic groups	456	320 (70.2)	136 (29.8)		
**Marital status**					
Married or living as a couple	1,133	661 (58.3)	472 (41.7)	0.87, 0.69 to 1.09	.216
vs. Single, widowed, divorced, or separated	755	434 (57.5)	321 (42.5)		
**Highest level of education**					Overall: **<.0005**
Other levels	199	116 (58.3)	83 (41.7)	2.32, 1.46 to 3.68	**<.0005**
Approximately Level 4	507	219 (43.2)	288 (56.8)	4.17, 2.75 to 6.33	**<.0005**
Approximately Level 1, 2, or 3	890	532 (59.8)	358 (40.2)	2.19, 1.52 to 3.15	**<.0005**
vs. No formal qualifications or don't know	292	228 (78.1)	64 (21.9)		
**Social class grade**					Overall: **.050**
Grade A or B	386	170 (44.0)	216 (56.0)	1.54, 1.09 to 2.17	**.014**
Grade C1 or C2	931	542 (58.2)	389 (41.8)	1.19, 0.91 to 1.55	.201
vs. Grade D or E	571	383 (67.1)	188 (32.9)		
**Personal diagnosis of cancer**					
Yes	98	49 (50.0)	49 (50.0)	1.10, 0.68 to 1.80	.690
vs. No	1,790	1,046 (58.4)	744 (41.6)		
**Knows someone with cancer**					
Yes	1,113	562 (50.5)	551 (49.5)	1.50, 1.19 to 1.89	**.001**
vs. No or don't know	775	533 (68.8)	242 (31.2)		
**Previously read a screening leaflet**					
Yes	1,058	535 (50.6)	523 (49.4)	1.27, 0.97 to 1.66	.089
vs. No or not sure	830	560 (67.5)	270 (32.5)		
**Previously read an NHS screening website**					
Yes	324	125 (38.6)	199 (61.4)	1.45, 1.07 to 1.97	**.016**
vs. No or not sure	1,564	970 (62.0)	594 (38.0)		
**Discussed screening with doctor/nurse**					
Yes	624	276 (44.2)	348 (55.8)	1.34, 1.03 to 1.74	**.030**
vs. No	1,264	819 (64.8)	445 (35.2)		
**Previously read or heard similar information**					
Yes	662	266 (40.2)	396 (59.8)	2.30, 1.81 to 2.94	**<.0005**
vs. No or not sure	1,226	829 (67.6)	397 (32.4)		
**Endorsed any term(s) as making sense**					
Yes	1,051	426 (40.5)	625 (59.5)	4.92, 3.94 to 6.17	**<.0005**
No	837	669 (79.9)	168 (20.1)		
**Perceived clarity of information**					Overall: .534
Extremely clear	170	87 (51.2)	83 (48.8)	0.85, 0.52 to 1.40	.525
Very clear	636	339 (53.3)	297 (46.7)	0.97, 0.65 to 1.43	.860
Moderately clear	685	424 (61.9)	261 (38.1)	0.78, 0.53 to 1.16	.225
Slightly clear	206	130 (63.1)	76 (36.9)	0.92, 0.57 to 1.49	.745
vs. Not at all clear	191	115 (60.2)	76 (39.8)		
		**Mean (SD)**			
**Age (in years)**	1,888	43.8 (15.7)	43.3 (15.8)	0.99, 0.98 to 1.00	**.003**

N.B. Adjusted ORs and 95% CIs are relative to a stated reference category except age, which is per unit increase; p-values <.05 are in bold; all predictor variables are included in the model

For peer review only

Table 4 – Number of participants (and percentages and 95% CIs) endorsing i) each number of possible terms and ii) each specific term as making sense to them as a label

		Number of terms endorsed as making sense as a way of describing the text: n, %, 95% CIs							
**Screening information**	Total	0	1	2	3	4	5	6	7
Breast	n (%)	429 (43.5)	76 (7.7)	86 (8.7)	74 (7.5)	67 (6.8)	58 (5.9)	42 (4.3)	155 (15.7)
n=987	95% CI	40.4 to 46.6	6.2 to 9.5	7.1 to 10.6	6.0 to 9.3	5.3 to 8.5	4.5 to 7.5	3.1 to 5.7	13.5 to 18.1
Prostate	n (%)	408 (45.3)	59 (6.5)	73 (8.1)	68 (7.5)	66 (7.3)	48 (5.3)	37 (4.1)	142 (15.8)
n=901	95% CI	42.1 to 48.5	5.1 to 8.3	6.5 to 10.0	6.0 to 9.4	5.8 to 9.2	4.0 to 6.9	3.0 to 5.6	13.5 to 18.2
Total	n (%)	837 (44.3)	135 (7.2)	159 (8.4)	142 (7.5)	133 (7.0)	106 (5.6)	79 (4.2)	297 (15.7)
N=1,888	95% CI	42.1 to 46.6	6.1 to 8.4	7.2 to 9.7	6.4 to 8.8	6.0 to 8.3	4.6 to 6.7	3.4 to 5.2	14.1 to 17.4

		"Which terms make sense as a way of describing this risk of a screening test?": n, %, 95% CIs						
**Screening information**	Total	Overdiagnosis	Overdetection	Unnecessary diagnosis	Overtreatment	Unnecessary treatment	False positive diagnosis	False positive test results
Breast	n (%)	334 (33.8)	275 (27.9)	349 (35.4)	336 (34.0)	409 (41.4)	324 (32.8)	338 (34.2)
n=987	95% CI	30.9 to 36.8	25.1 to 30.7	32.4 to 38.4	31.1 to 37.0	38.4 to 44.5	30.0 to 35.8	31.3 to 37.2
Prostate	n (%)	291 (32.3)	247 (27.4)	307 (34.1)	280 (31.1)	354 (39.3)	320 (35.5)	330 (36.6)
n=901	95% CI	29.3 to 35.4	24.6 to 30.4	31.0 to 37.2	28.1 to 34.2	36.1 to 42.5	32.4 to 38.7	33.5 to 39.8
Total	n (%)	625 (33.1)	522 (27.6)	656 (34.7)	616 (32.6)	763 (40.4)	644 (34.1)	668 (35.4)
N=1,888	95% CI	31.0 to 35.3	25.7 to 29.7	32.6 to 36.9	30.5 to 34.8	38.2 to 42.6	32.0 to 36.3	33.2 to 37.6

For peer review only

Table 5 – Endorsed one or more vs. no terms as making sense: descriptive statistics, adjusted ORs, 95% CIs, and p-values for variables in the multivariable binary logistic regression model

		Endorsed no vs. at least one term as making sense: n (%)		Adjusted OR, 95% CI	p-value
**Characteristic**	**Total (N=1,888)**	**No term (n=837; 44.3%)**	**At least one term (n=1,051; 55.7%)**	**At least one term endorsed (vs. No terms endorsed)**	
**Overdiagnosis information**					
Breast screening text	987	429 (43.5)	558 (56.5)	1.16, 0.95 to 1.42	.148
vs. Prostate screening text	901	408 (45.3)	493 (54.7)		
**Gender**					
Male	879	380 (43.2)	499 (56.8)	1.40, 1.13 to 1.74	**.002**
vs. Female	1,009	457 (45.3)	552 (54.7)		
**Ethnicity**					
White British	1,432	592 (41.3)	840 (58.7)	1.25, 0.97 to 1.61	.080
vs. Other ethnic groups	456	245 (53.7)	211 (46.3)		
**Marital status**					
Married or living as a couple	1,133	500 (44.1)	633 (55.9)	1.05, 0.85 to 1.30	.671
vs. Single, widowed, divorced, or separated	755	337 (44.6)	418 (55.4)		
**Highest level of education**					Overall: .556
Other levels	199	88 (44.2)	111 (55.8)	1.21, 0.80 to 1.82	.374
Approximately Level 4	507	190 (37.5)	317 (62.5)	1.27, 0.88 to 1.84	.204
Approximately Level 1, 2, or 3	890	396 (44.5)	494 (55.5)	1.24, 0.91 to 1.70	.169
vs. No formal qualifications or don't know	292	163 (55.8)	129 (44.2)		
**Social class grade**					Overall: .574
Grade A or B	386	137 (35.5)	249 (64.5)	1.09, 0.78 to 1.51	.617
Grade C1 or C2	931	420 (45.1)	511 (54.9)	0.94, 0.74 to 1.19	.604
vs. Grade D or E	571	280 (49.0)	291 (51.0)		
**Personal diagnosis of cancer**					
Yes	98	37 (37.8)	61 (62.2)	1.08, 0.68 to 1.74	.741
vs. No	1,790	800 (44.7)	990 (55.3)		
**Knows someone with cancer**					
Yes	1,113	427 (38.4)	686 (61.6)	1.35, 1.09 to 1.68	**.005**
vs. No or don't know	775	410 (52.9)	365 (47.1)		
**Previously read a screening leaflet**					
Yes	1,058	416 (39.3)	642 (60.7)	1.18, 0.91 to 1.52	.206
vs. No or not sure	830	421 (50.7)	409 (49.3)		
**Previously read an NHS screening website**					
Yes	324	109 (33.6)	215 (66.4)	1.01, 0.75 to 1.37	.955
vs. No or not sure	1,564	728 (46.5)	836 (53.5)		
**Discussed screening with doctor/nurse**					
Yes	624	217 (34.8)	407 (65.2)	1.25, 0.97 to 1.61	.091
vs. No	1,264	620 (49.1)	644 (50.9)		
**Previously read or heard similar information**					
Yes	662	224 (33.8)	438 (66.2)	1.18, 0.93 to 1.50	.185
vs. No or not sure	1,226	613 (50.0)	613 (50.0)		
**Previously encountered any term(s)**					
Yes	1,051	168 (21.2)	625 (78.8)	4.88, 3.89 to 6.11	**<.0005**
No	837	669 (61.1)	426 (38.9)		
**Perceived clarity of information**					Overall: .148
Extremely clear	170	67 (39.4)	103 (60.6)	1.36, 0.85 to 2.17	.204
Very clear	636	255 (40.1)	381 (59.9)	1.38, 0.96 to 1.98	.083
Moderately clear	685	308 (45.0)	377 (55.0)	1.39, 0.97 to 1.98	.073
Slightly clear	206	109 (52.9)	97 (47.1)	0.99, 0.64 to 1.53	.953
vs. Not at all clear	191	98 (51.3)	93 (48.7)		
		**Mean (SD)**			
**Age (in years)**	1,888	43.4 (15.6)	43.7 (15.8)	1.00, 0.99 to 1.00	.262

N.B. Adjusted ORs and 95% CIs are relative to a stated reference category except age, which is per unit increase; p-values <.05 are in bold; all predictor variables are included in the model

For peer review only

DISCUSSION

This study aimed to address a gap in the literature regarding how best to communicate the concept of overdiagnosis (8). We investigated whether people are familiar with terms used to describe overdiagnosis or related concepts, and whether these made sense as labels for widely used descriptions. We found that a majority of participants (58.0%) had never encountered any of the assessed terms before and a large proportion (44.3%) did not rate any term as making sense as a suitable label for either of two widely used descriptions of the concept. In addition, no specific term(s) emerged as being notably more familiar or applicable.

A previous similar study found that only 30.0% of the UK general public (aged 50-70) had previously encountered the specific term, 'overdiagnosis' (9), which is broadly comparable to the 21.8% who reported having encountered it in this study. Our findings are also consistent with those of a previous study that was part of redesign of the information materials for the NHS Breast Screening Programme (20). Women were interviewed with the aim of finding ways to communicate the concept of overdiagnosis: participants generally struggled to understand it, regardless of whether it was labelled as 'overdiagnosis' or 'overtreatment'.

Terminology can have a role in transferring information to lay individuals as part of a description of technical concepts (11,21). However, the main implication of our results is that currently there may only be limited value in using any of the labels that we tested. Terms used were neither familiar nor rated as particularly relevant in this large, diverse sample, meaning that they may be confusing, unintuitive, and hard for people to memorise and recall. Even the most technically appropriate terms (e.g. 'overdiagnosis' and 'overdetection') were not endorsed as making sense more frequently than suggested alternative terminology (e.g. 'unnecessary diagnosis) or more inaccurate and potentially problematic terminology (e.g. 'false positive diagnosis'). To some

For peer review only

extent, the limited familiarity and low levels of endorsement at present supports UK policy decisions not to use a specific term in the revised breast screening information leaflet (20). Furthermore, shortly before the start of recruitment, an updated NHS information leaflet regarding PSA screening was published, with additional detail on overdiagnosis and overtreatment (22); this also omitted any specific descriptive label.

One unexpected finding was that the second most common number of terms that were familiar or endorsed was all seven (12.3% and 15.7%, respectively). This may be due to differences between terms being subtle: members of the public may not recall exactly which terms they had encountered but felt that broader concepts like diagnosis and treatment were familiar.

We found that participants with a lower level of education, who were not White British, were older, or (possibly) were from a lower social class grade were less likely to have previously encountered any term(s), suggesting that these groups may be particularly poorly served by the use of any specific terminology. Unsurprisingly, those who had previous exposure to cancer (i.e. by knowing someone with a diagnosis) and those who had encountered potentially relevant information (e.g. those who had previously discussed screening with a doctor or nurse) were more likely to have encountered any term(s). In some respects, this latter finding was surprising because although medical staff might be expected to discuss overdiagnosis with patients, there is evidence that the topic is explained only rarely (23). Those who knew someone with cancer were also more likely to endorse at least one term as making sense. It is unclear why males were more likely to endorse term(s) as making sense. This may warrant further research (possible explanations may include a degree of over-confidence in men or under-confidence in women), although this should be considered in the context of small absolute differences observed here (56.8% vs. 54.7% endorsing at least one term).

For peer review only

Continuing communication efforts mean that these results may change over time: it is plausible that ongoing campaigns (3-5) will result in the public becoming increasingly familiar with the concept and terminology, having a better understanding of the differences between similar terms (e.g. seeing ‘false positives’ as distinct from ‘overdiagnosis’), and being more likely to endorse particular terms as applicable. However, present attempts to communicate overdiagnosis (e.g. as part of screening invitations or mainstream media stories) may be more effective if explicit descriptions are used, such as adapted forms of information from the Breast Screening Programme. This has undergone an extensive design process (20) and is more likely to be rated as clearer than the equivalent from the Prostate Cancer Risk Management Programme. However, fewer than half of participants (46.6%) rated it as very or extremely clear, suggesting that future research could be used to improve it further (e.g. by providing additional detail; 15).

This study has limitations. The data collection method used by the survey company meant that demographic characteristics used to ensure population-representativeness were not exhaustive (e.g. they omitted ethnicity and education) and response rates were not available. The list of terms tested was also not exhaustive; there may be suitable terminology that we did not assess. However, we found very similar levels of endorsement for terms that used several variations on the themes of diagnosis, treatment, and false positives, providing some evidence that any superior terms would have to be quite different. In addition, as noted in our previous study, descriptions of overdiagnosis were brief and presented outside the context of the original written information about screening from which they were adapted; comprehension may be different (either better or worse) in the presence of additional detail (15); this warrants further research.

Conclusions

For peer review only

Alternative terminology to 'overdiagnosis' may help increase awareness and understanding of the concept among individuals who may face healthcare decisions that would put them at risk. These results suggest that, at present, using specific terms would have limited benefits and may be less well suited to particular groups (e.g. less educated or non-White British individuals). It may currently be more effective to refer to the concept via more explicit descriptions. Previous research indicates that information from the NHS Breast Screening Programme may be a relatively effective template for this purpose. However, future research could explore scope for improvement.

Contributors: AG, CR, and JW conceived and designed the study. AG analysed the data. AG, CR, and JW participated in the interpretation of results. AG, CR, and JW drafted the manuscript, participated in critical revision, and approved the final version.

Funding: This work was supported by a programme grant from Cancer Research UK awarded to Prof Jane Wardle [C1418/A14134]. Dr Jo Waller is supported by a Career Development Fellowship from Cancer Research UK [C7492/A17219]. Cancer Research UK was not involved in the design of this study; the collection, analysis, or interpretation of the results; in the writing of the manuscript; or in the decision to submit for publication.

Competing interests: None declared.

Patient consent: Obtained.

Data sharing: No additional data are available.

REFERENCES

1. Heath I. Overdiagnosis: when good intentions meet vested interests — an essay by Iona

For peer review only

Heath. Br Med J. 2013;347:f6361.

2. Moynihan R, Doust J, Henry D. Preventing overdiagnosis: how to stop harming he healthy. Br Med J. 2012;344:e3502.

3. Glasziou P, Moynihan R, Richards T. Godlee F. Too much medicine; too little care. Br Med J. 2013;347:f4247.

4. Malhotra A, Maughan D, Ansell J, et al. Choosing Wisely in the UK: the Academy of Medical Royal Colleges' initiative to reduce the harms of too much medicine. Br Med J. 2015;350:h2308.

5. Calderwood C. Chief Medical Officer's annual report 2014-15. 2016. http://www.gov.scot/Publications/2016/01/3745/0

6. Hersch J, Jansen J, Barratt A, et al. Women's views on overdiagnosis in breast cancer screening: a qualitative study. Br Med J. 2013;346:f158.

7. Waller J, Douglas E, Whitaker KL, Wardle J. Women's responses to information about overdiagnosis in the UK breast cancer screening programme: a qualitative study. BMJ Open. 2013;3:e002703.

8. McCaffery KJ, Jansen J, Scherer LD, et al. Walking the tightrope: communicating overdiagnosis in modern healthcare. Br Med J. 2016;352:i348.

9. Ghanouni A, Meisel SF, Renzi C, Wardle J, Waller J. Public definitions of the term 'overdiagnosis' in the UK. BMJ Open. 2016;e010723.

10. Moynihan R, Nickel B, Hersch J, et al. What do you think overdiagnosis means? A

For peer review only

qualitative analysis of responses from a national community survey of Australians. BMJ Open. 2015;5:e007436.

11. Hersch J, Jansen J, Barratt A, et al. Overdetection in breast cancer screening: development and preliminary evaluation of a decision aid. BMJ Open. 2014;4:e006016.

12. Moynihan R, Nickel B, Hersch J, et al. Public opinions about overdiagnosis: a national community survey. PLoS One. 2015;10:e0125165.

13. NHS Breast Screening Programme. NHS Breast Screening: Helping you decide. 2013. Available from: https://www.gov.uk/government/uploads/system/uploads/attachment_data/file/440798/nhsbsp.pdf

14. Burford D, Austoker J, Kirby M. PSA (prostate specific antigen) testing for prostate cancer. An information sheet for men considering a PSA test. 2009.

15. Ghanouni A, Renzi C, McBride E, Waller J. Comparing perceived clarity of information on overdiagnosis used for breast and prostate cancer screening in England: an experimental survey. BMJ Open. 2017;7:e015955.

16. National Readership Survey. Social grade. http://www.nrs.co.uk/nrs-print/lifestyle-and-classification-data/social-grade/ Accessed: 1st December 2017.

17. Weinstein ND. The precaution adoption process. Heal Psychol. 1988;7:355–86.

18. Willis GB , Artino AR . What do our respondents think we're asking? using cognitive interviewing to Improve Medical Education surveys. J Grad Med Educ 2013;5:353–6.

For peer review only

19. Office of National Statistics. 2011 Census: Key Statistics for England and Wales, March 2011. 2012 http://www.ons.gov.uk/ons/rel/census/2011-census/key-statistics-for-local-authorities-in-england-and-wales/stb-2011-census-key-statistics-for-england-and-wales.html (accessed 28 Nov 2016).

20. Forbes LJ, Ramirez AJ, Expert group on Information about Breast Screening. Offering informed choice about breast screening. J Med Screen. 2014;21:194–200.

21. Fage-Butler AM, Nisbeth Jensen M. Medical terminology in online patient-patient communication: evidence of high health literacy? Heal Expect. 2016;19:643–53.

22. Public Health England. PSA testing and prostate cancer: advice for well men aged 50 and over. 2016. Available from: https://www.gov.uk/government/uploads/system/uploads/attachment_data/file/509191/Patient_info_sheet.pdf

23. Wegwarth O, Gigerenzer G. Less is more: overdiagnosis and overtreatment: evaluation of what physicians tell their patients about screening harms. JAMA Intern Med. 2013;173:2086-7.

For peer review only

BMJ Open

Improving public understanding of 'overdiagnosis' in England: a population survey assessing familiarity with possible terms for labelling the concept and perceptions of appropriate terminology

Journal:	*BMJ Open*
Manuscript ID	bmjopen-2017-021260.R2
Article Type:	Research
Date Submitted by the Author:	17-Apr-2018
Complete List of Authors:	Ghanouni, Alex; University College London, Research Department of Behavioural Science and Health<br>Renzi, Cristina; UCL, Research Department of Behavioural Science and Health<br>Waller, Jo; UCL, Research Department of Behavioural Science and Health
**Primary Subject Heading**:	Communication
Secondary Subject Heading:	Epidemiology, Public health, Health services research
Keywords:	EPIDEMIOLOGY, PUBLIC HEALTH, Health policy < HEALTH SERVICES ADMINISTRATION & MANAGEMENT

SCHOLARONE™
Manuscripts

For peer review only

FULL TITLE PAGE

Title: Improving public understanding of 'overdiagnosis' in England: a population survey assessing familiarity with possible terms for labelling the concept and perceptions of appropriate terminology

Manuscript type: Research article

Authors:

Alex Ghanouni[a] a.ghanouni@ucl.ac.uk

Cristina Renzi[a] c.renzi@ucl.ac.uk

Jo Waller[ab] j.waller@ucl.ac.uk | Tel: +44 (0)20 7679 5958 | Fax: +44 (0)20 7679 8354

Affiliations:

[a]Research Department of Behavioural Science and Health, University College London, Gower Street, London, WC1E 6BT, United Kingdom

[b]Corresponding author

Word count: Abstract: 299/300; Body: 3,493/4,000; References: 24; Tables or figures: 5

For peer review only

ABSTRACT

Objectives: Communicating the concept of 'overdiagnosis' to lay individuals is challenging, partly because the term itself is confusing. This study tested whether alternative descriptive labels may be more appropriate.

Design: Questionnaire preceded by a description of overdiagnosis.

Setting: Home-based, computer-assisted face-to-face survey.

Participants: 2,111 adults aged 18 to 70 years in England recruited using random location sampling by a survey company. Data from 1,888 participants were analysed after exclusions due to missing data.

Interventions: Participants were given one of two pieces of text describing overdiagnosis, allocated at random, adapted from NHS breast and prostate cancer screening leaflets.

Primary and secondary outcome measures: Main outcomes were which of several available terms (e.g. 'overdetection') participants had previously encountered and which they endorsed as applicable labels for the concept described. Demographics and previous exposure to screening information were also measured. Main outcomes were summarised with descriptive statistics. Predictors of previously encountering at least one term, or endorsing at least one as making sense, were assessed using binary logistic regression.

Results: 58.0% of participants had not encountered any suggested term; 44.0% did not endorse any as applicable labels. No term was notably familiar; the proportion of participants who had previously encountered each term ranged from 15.9 to 28.3%. Each term was only endorsed as applicable by a minority (range: 27.6% to 40.4%). Notable predictors of familiarity included

For peer review only

education, age, and ethnicity; participants were less likely to have encountered terms if they were older, not White British or had less education, Findings were similar for both pieces of information.

Conclusions: Familiarity with suggested terms for overdiagnosis and levels of endorsement were low and no clear alternative labels for the concept were identified, suggesting that changing terminology alone would do little to improve understanding, particularly for some population groups. Explicit descriptions may be more effective.

Keywords: Decision Making; Surveys and Questionnaires; Early Detection of Cancer; Mass Screening; Medical Overuse

Strengths and limitations of this study

- This study assessed (predictors of) familiarity and perceived appropriateness of a broad range of possible terms for the concept of 'overdiagnosis' among a large, representative sample of the general public in England.
- The concept was described based on information that was widely used by the National Health Service in England, maintaining generalisability.
- The demographic criteria used to achieve population-representativeness were not comprehensive and response rates were not available.
- The list of possible terms was also not exhaustive; more suitable alternative labels may have been omitted.
- Results may be time-dependent: ongoing communication initiatives may change perceptions of appropriate terms for the concept of 'overdiagnosis'.

For peer review only

INTRODUCTION

There is growing concern among healthcare providers and policymakers about the potential for medical tests to detect asymptomatic disease that would never have become clinically apparent or resulted in death (1). ‘Overdiagnosis’ can harm, primarily via the subsequent risks and costs associated with unnecessary treatment but also via opportunity costs to a healthcare service due to overuse of scarce resources (2). Various initiatives have aimed to propagate understanding of the concept among patients and the public so that they can make better informed decisions about their health (e.g. 3–5). However, previous studies have found that the concept is challenging to communicate to lay people (e.g. in the context of breast cancer screening; 6,7).

Little is known about how to improve communication of overdiagnosis (8). One barrier is that the term ‘overdiagnosis’ itself may be confusing and counterintuitive. For example, a previous study in the UK asked members of the public to give their interpretation of what the word meant; a large proportion of participants gave definitions that did not match the concept but resembled other similar words (e.g. ‘providing an incorrect diagnosis’ since 'overdiagnosis' resembles 'misdiagnosis; 9). Equivalent findings have been reported by a previous survey in Australia (10). In addition, a focus group study undertaken as part of the development of a decision aid for breast screening found that 'overdiagnosis' was not understood intuitively and that ‘overdetection’ may be clearer (11).

These findings suggest that the term ‘overdiagnosis’ does not clearly reflect its intended meaning for the general public and that other terms (such as 'overdetection') may be more appropriate. Hence, this study asked a large sample of the public in England to appraise descriptions of the concept and tested which of several possible terms people thought made

For peer review only

sense as labels (i.e. endorsement). Alternatives included 'overdetection' or 'unnecessary diagnosis' (11) as well as terms related to unnecessary therapy (e.g. 'overtreatment') and terms that were less technically accurate but still had the potential to be seen as applicable (e.g. 'false positives'; 9, 10). A limitation to the utility of 'overdiagnosis' as a label may be the currently low level of public awareness of the term (9, 12). Consequently, this study also measured which terms people had encountered before (i.e. familiarity) to test whether any alternatives were notably more familiar. We also explored whether participant characteristics were associated with having previously encountered these terms, or with endorsing them as making sense, as this may aid targeted communication efforts.

Information was derived from materials that have been widely used by the NHS Breast Screening Programme (13) and the Prostate Cancer Risk Management Programme (14). The study was carried out as part of Wave 3 of the Attitudes, Behaviour and Cancer UK Survey (ABACUS). Other studies arising from this survey have also been published (e.g. 15).

METHODS

Design

Institutional approval was granted by the University College London Research Ethics Committee (5771/002). The focus of the present study was on appropriate terminology for widely used descriptions of overdiagnosis. However, the ABACUS survey was a broader piece of work that also compared the perceived clarity of different forms of overdiagnosis information. Hence, elements of the design and measures have also been reported elsewhere (15). Face-to-face computer-assisted interviews were conducted by TNS (a market research company) as part of a weekly omnibus survey and took place in participants' homes between April and May 2016. Participants were informed:

For peer review only

"We would now like to ask you some questions about leaflets on health-related topics. The NHS offers people a variety of screening tests to check for illnesses before symptoms have appeared. People offered an NHS screening test are often given a leaflet that explains the risks and benefits of having the test. The leaflet is either posted or given out by a doctor or nurse."

Participants then received information on overdiagnosis adapted from written material used by the NHS in England as part of either i) the Breast Screening Programme or ii) the Prostate Cancer Risk Management Programme as they existed in February 2016 (neither of which used a specific label), allocated at random in a 1:1 ratio:

"The test can find an illness that would never have caused a person harm. Some people will be diagnosed and treated for an illness that would never otherwise have been found and would not have become life-threatening." From the breast screening information leaflet (13)

"The test may make you worry by finding an illness that may never cause any symptoms or shorten your life." From the prostate screening information leaflet (14)

The Breast Screening Programme in England offers triennial mammography to all women between the ages of 50 to 70 years and registered with a General Practitioner. The Prostate Screening Risk Management Programme allows men aged 50 years or older to have a Prostate Specific Antigen test by requesting it from their GP after discussing the risks and benefits. References to cancer were removed so that findings could be generalised to beyond these two specific screening contexts.

Participants

Participants were members of the public in England aged 18 to 70 years. National representativeness was sought via a two-stage process. In the first instance, random location

For peer review only

sampling was carried out using the Postcode Address File and Census statistics. Within each selected location, participant quotas were set based on demographic characteristics (i.e. age, gender, employment status, and presence of children in the home).

Measures

Demographics: Relevant measures consisted of age, gender, ethnicity, social grade (16), marital status, highest level of education obtained, personal history of cancer (*"Have you ever been diagnosed with cancer?"*; *"yes"* or *"no"*), and whether anyone they know had been diagnosed with cancer (*"Has anyone close to you ever been diagnosed with cancer?"*; *"yes"*, *"no"*, or *"don't know"*).

Familiarity with screening information: Participants were asked whether they had ever previously received information about screening (for cancer or other illnesses) via i) reading a leaflet from the NHS, ii) reading an NHS website, and iii) talking with a doctor or nurse (response options: *"yes"*, *"no"*, *"not sure"*).

If participants were eligible for cancer screening and had not been diagnosed with the target disease, they were asked about their previous participation (e.g. only women aged 47-70 years without a breast cancer diagnosis were asked about breast screening participation) via items designed using the Precaution Adoption Process Model (PAPM; 17), a stage theory of protective health behaviour. This measures whether participants have heard of a particular type of screening, whether they have heard of a type of screening but have never been invited, whether they have been invited but never participated, whether they have participated but not consistently, and whether they have participated consistently. Eligibility criteria and exact question wording for assessing experience have been reported elsewhere (15).

Perceived clarity of information: After reading the information allocated at random, participants were asked, *"How clear do you find this description of a risk of the test?"* with available responses of *"extremely clear"*, *"very clear"*, *"moderately clear"*, *"slightly clear"*, and *"not at all clear"*. This was followed by a question on whether they had read or heard similar information about a screening test before (possible responses were: *"yes"*, *"no"*, and *"not sure"*).

Main outcomes: The first main outcome measure (endorsement) consisted of *"Does this term make sense to you as a way of describing this risk of a screening test?"*, followed by seven terms: *"overdiagnosis"*, *"overdetection"*, *"unnecessary diagnosis"*, *"overtreatment"*, *"unnecessary treatment"*, *"false positive diagnosis"*, and *"false positive test results"*[1] (responses: *"yes"*, *"no"*). Suggested terms were based on previous literature (e.g. 11) and discussion among the research team.

The second outcome measure (familiarity) was: "Have you ever seen or heard any of the previous seven terms before today?" and if participants responded *"yes"*, they were asked which out of the previous terms (responses: *"yes"*, *"no"*, *"not sure"*).

Ancillary measures: The measures used in this study were part of the broader ABACUS survey. Most of the items in this study were presented at the start of the survey and so the effects of priming from unrelated items were expected to be minimal. In order, the full survey included: questions on decision-making style and previous exposure to cancer screening information,

[1] Participants were also asked, *"Can you think of any other terms to describe this risk that would make sense to you?"*. However, only approximately 60 responses suggesting potentially applicable terms were recorded across all participants (e.g. *"oversensitivity"*, *"unknown diagnosis"*), of which most were mentioned just once and so no further analysis was attempted.

For peer review only

information on overdiagnosis adapted from screening leaflets, questions on perceived clarity of this information, previous exposure to similar information, the main study outcomes, and help-seeking behaviour in relation to cancer screening, followed by more questions on self-rated health, questions on cancer diagnoses, perceived cancer risk, screening behaviour in relation to each of the applicable programmes (cervical, breast, bowel, prostate), and educational level.

Public Involvement

Public involvement consisted of input into the design of the study and development of measures via pilot testing of the survey. This comprised two stages: first, a series of telephone cognitive interviews (18) was used to determine whether items could be understood by lay people (n=11) and that the survey was not overly burdensome. Participants were asked for feedback on any items they found difficult to understand or answer and the survey was revised accordingly. Second, a web-based version of the survey was pilot-tested with 431 participants. There are currently no plans to disseminate the results to study participants.

Analysis

Participants were excluded if they declined to answer any of the measures included in this study. Ethnicity and marital status were dichotomised into “White British” and “other ethnic groups”, and “single, widowed, divorced or separated” and “married or living as a couple”, respectively. Social class grade was categorised as “grades A or B”, “grades C1 or C2”, and “grades D or E”. Education was coded based on Levels 1-4 from the Office of National Statistics (19) or “Other” for non-ordinal levels of education such as professional qualifications.

Sample characteristics were summarised with descriptive statistics. We report the percentages of participants (with binomial 95% confidence intervals) who had i) previously encountered and

For peer review only

ii) endorsed each possible number of terms (from 0 to 7 in both cases), and the percentages of participants who had previously encountered and endorsed each specific term.

Responses to i) and ii) were also recoded into “previously seen or heard one or more terms” (familiarity) and “endorsed one or more terms as making sense to them” (endorsement) for further evaluation e.g. “was familiar with at least one term” vs. “was not familiar” (including responses of “not sure”). Two exploratory logistic regression models tested the null hypothesis that predictor variables were unrelated to either of these outcomes. In both models, predictor variables consisted of demographic characteristics, familiarity with screening information, the overdiagnosis information condition (breast vs. prostate), and perceived clarity. In addition, each model included the outcome from the other as a predictor (e.g. the endorsement model included familiarity as a potential predictor variable). Variance Inflation Factors were small (all <2.969 and <2.972, respectively), indicating that (multi)collinearity was limited and a Box-Tidwell procedure found little evidence to suggest that the age variable violated the assumption of linearity (p-values: .110 and .508). Adjusted odds-ratios (ORs), accompanying 95% CIs, and p-values for having previously seen or heard one or more terms before and endorsing one or more term as making sense are reported alongside descriptive statistics.

RESULTS

Participant characteristics

After excluding 223 participants with missing data, 1,888 cases were analysed. Sample characteristics are presented in Table 1. Mean age was 43.6 years (standard deviation: 15.7).

Familiarity with terms

For peer review only

Table 2 illustrates that the majority of participants (58.0%) were not familiar with any of the listed terms. However, the second most common number of terms recognised as familiar was all seven (12.3%). The percentages of participants previously encountering between one and six terms ranged from 1.1% to 9.4%. Table 2 also shows that the percentages of participants who had encountered each specific term ranged from 15.9% (*"overdetection"*) to 28.3% (*"false positive test results"*).

Table 3 reports predictors of familiarity with at least one term. Among demographic characteristics, there was strong evidence against the null hypothesis for ethnicity, education, and age: White British participants were more likely to be familiar with at least one term compared with other ethnic groups. Participants with a higher (or 'other') level of education were also more likely to be familiar with one or more terms compared with participants who had no formal qualifications (or did not know). Older participants were less likely to be familiar with one or more terms. Participants were also more likely to be familiar with one or more terms if they knew someone with cancer, had previously read an NHS website about screening, discussed screening with a doctor or nurse, had previously read or seen similar information, or endorsed one or more terms as making sense as labels.

Finally, there was moderate evidence against the null hypothesis for an association with social class grade with participants in grades A or B being more likely to be familiar with at least one term compared with participants in grades D or E.

Terms endorsed as making sense

Table 4 shows that participants most commonly did not endorse any terms as making sense (44.3%). As with results for familiarity, the second most common number of terms endorsed was all seven (15.7%). The most commonly endorsed term was *"unnecessary treatment"* (40.4% of

For peer review only

participants in the sample endorsed this term overall; 41.4% and 39.3% after being given the breast and prostate information, respectively). Endorsement of other terms ranged from 27.9% (*"overdetection"*) to 35.4% (*"unnecessary diagnosis"*) for information from breast screening and 27.4% (*"overdetection"*) to 36.6% (*"false positive test results"*) for information from prostate screening.

Other than familiarity (Table 3), the logistic regression analysis of predictors of endorsement only showed strong evidence against the null hypothesis for gender and knowing someone with cancer: males and participants who knew someone diagnosed with cancer were more likely to endorse at least one term as making sense (Table 5).

For peer review only

Table 1 – Characteristics of the sample

	Total N=1,888	
**Characteristic**	**n**	**(%)**
**Overdiagnosis information**		
Breast screening text	987	(52.3)
Prostate screening text	901	(47.7)
**Gender**		
Male	879	(46.6)
Female	1,009	(53.4)
**Ethnicity**		
White British	1,432	(75.8)
Other ethnic groups	456	(24.2)
**Marital status**		
Married or living as a couple	1,133	(60.0)
Single, widowed, divorced, or separated	755	(40.0)
**Highest level of education**[1]		
No formal qualifications	281	(14.9)
Approximately Level 1, 2, or 3	890	(47.1)
Approximately Level 4	507	(26.9)
Other	199	(10.5)
Don't know/Not sure	11	(0.6)
**Social class grade**[2]		
Grade A or B	386	(20.4)
Grade C1 or C2	931	(49.3)
Grade D or E	571	(30.2)
**Personal diagnosis of cancer**		
Yes	98	(5.2)
No	1,790	(94.8)
**Knows someone with cancer**		
Yes	1,113	(59.0)
No	771	(40.8)
Don't know/Not sure	4	(0.2)
**Any previous cervical screening experience**		
Yes	652	(34.5)
No	226	(12.0)
Not eligible	1,010	(53.5)
**Any previous breast screening experience**		
Yes	317	(16.8)
No	83	(4.4)
Not eligible	1,488	(78.8)
**Any previous bowel screening experience**		
Yes	293	(15.5)
No	181	(9.6)
Not eligible	1,414	(74.9)
**Any previous prostate screening experience**		
Yes	77	(4.1)
No	344	(18.2)
Not eligible	1,467	(77.7)
**Previously read a screening leaflet**		
Yes	1,058	(56.0)
No	809	(42.8)
Don't know/Not sure	21	(1.1)
**Previously read an NHS screening website**		
Yes	324	(17.2)
No	1,549	(82.0)
Don't know/Not sure	15	(0.8)
**Discussed screening with doctor/nurse**		
Yes	624	(33.1)
No	1,251	(66.3)
Don't know/Not sure	13	(0.7)
**Previously read or heard similar information**		
Yes	662	(35.1)
No	1,185	(62.8)
Don't know/Not sure	41	(2.2)

For peer review only

**Perceived clarity of information**		
Extremely clear	170	(9.0)
Very clear	636	(33.7)
Moderately clear	685	(36.3)
Slightly clear	206	(10.9)
Not at all clear	191	(10.1)

[1] Level 1-3 qualifications include e.g. GCSEs and A Levels; Level 4 qualifications include degrees and higher degrees
[2] Social grades are based on occupation (e.g. Grade A includes managerial roles; Grade E includes casual workers)

For peer review only

Table 2 – Number of participants (and percentages and 95% CIs) previously encountering i) each number of possible terms and ii) each specific term. Participants receiving overdiagnosis information based on the Breast Screening programme or the Prostate Cancer Risk Management Programme were analysed separately.

		Number of terms previously encountered: n, %, 95% CIs							
**Screening information**	Total	0	1	2	3	4	5	6	7
Breast	n (%)	583 (59.1)	73 (7.4)	76 (7.7)	53 (5.4)	41 (4.2)	28 (2.8)	9 (0.9)	124 (12.6)
N=987	95% CI	56.0 to 62.1	5.9 to 9.2	6.2 to 9.5	4.1 to 6.9	3.0 to 5.5	1.9 to 4.0	0.5 to 1.7	10.6 to 14.7
Prostate	n (%)	512 (56.8)	64 (7.1)	101 (11.2)	47 (5.2)	37 (4.1)	19 (2.1)	12 (1.3)	109 (12.1)
n=901	95% CI	53.6 to 60.0	5.6 to 8.9	9.3 to 13.4	3.9 to 6.8	3.0 to 5.6	1.3 to 3.2	0.7 to 2.2	10.1 to 14.3
Total	n (%)	1,095 (58.0)	137 (7.3)	177 (9.4)	100 (5.3)	78 (4.1)	47 (2.5)	21 (1.1)	233 (12.3)
N=1,888	95% CI	55.8 to 60.2	6.2 to 8.5	8.1 to 10.8	4.4 to 6.4	3.3 to 4.1	1.9 to 3.3	0.7 to 1.7	10.9 to 13.9

		"Which of the following term or terms had you heard of before?": n, %, 95% CIs							
**Screening information**	Total	Not sure	Overdiagnosis	Overdetection	Unnecessary diagnosis	Overtreatment	Unnecessary treatment	False positive diagnosis	False positive test result
Breast	n (%)	7 (0.7)	215 (21.8)	163 (16.5)	214 (21.7)	225 (22.8)	280 (28.4)	243 (24.6)	270 (27.4)
N=987	95% CI	0.3 to 1.4	19.3 to 24.4	14.3 to 18.9	19.2 to 24.3	20.3 to 25.5	25.6 to 31.2	22.0 to 27.4	24.6 to 30.2
Prostate	n (%)	14 (1.6)	196 (21.8)	137 (15.2)	216 (24.0)	186 (20.6)	253 (28.1)	232 (25.7)	265 (29.4)
n=901	95% CI	0.9 to 2.5	19.2 to 24.5	13.0 to 17.7	21.3 to 26.8	18.1 to 23.4	25.2 to 31.1	23.0 to 28.7	26.5 to 32.4
Total	n (%)	21 (1.1)	411 (21.8)	300 (15.9)	430 (22.8)	411 (21.8)	533 (28.2)	475 (25.2)	535 (28.3)
N=1,888	95% CI	0.7 to 1.7	20.0 to 23.7	14.3 to 17.6	20.9 to 24.7	20.0 to 23.7	26.2 to 30.3	23.2 to 27.2	26.3 to 30.4

For peer review only

Table 3 – Familiarity with one or more vs. no terms: descriptive statistics, adjusted ORs, 95% CIs, and p-values for variables in the multivariable binary logistic regression model

		**Familiarity with no terms vs. at least one term: n (%)**		**Adjusted OR, 95% CI**	**p-value**
**Characteristic**	**Total (N=1,888)**	**No term (n=1,095; 58.0%)**	**At least one term (n=793; 42.0%)**	**At least one familiar term (vs. None)**	
**Overdiagnosis information**					
Breast screening text	987	583 (59.1)	404 (40.9)	0.87, 0.70 to 1.09	.224
vs. Prostate screening text	901	512 (56.8)	389 (43.2)		
**Gender**					
Male	879	534 (60.8)	345 (39.2)	1.00, 0.79 to 1.26	.998
vs. Female	1,009	561 (55.6)	448 (44.4)		
**Ethnicity**					
White British	1,432	775 (54.1)	657 (45.9)	2.14, 1.61 to 2.84	**<.0005**
vs. Other ethnic groups	456	320 (70.2)	136 (29.8)		
**Marital status**					
Married or living as a couple	1,133	661 (58.3)	472 (41.7)	0.87, 0.69 to 1.09	.216
vs. Single, widowed, divorced, or separated	755	434 (57.5)	321 (42.5)		
**Highest level of education**					Overall: **<.0005**
Other levels	199	116 (58.3)	83 (41.7)	2.32, 1.46 to 3.68	**<.0005**
Approximately Level 4	507	219 (43.2)	288 (56.8)	4.17, 2.75 to 6.33	**<.0005**
Approximately Level 1, 2, or 3	890	532 (59.8)	358 (40.2)	2.19, 1.52 to 3.15	**<.0005**
vs. No formal qualifications or don't know	292	228 (78.1)	64 (21.9)		
**Social class grade**					Overall: **.050**
Grade A or B	386	170 (44.0)	216 (56.0)	1.54, 1.09 to 2.17	**.014**
Grade C1 or C2	931	542 (58.2)	389 (41.8)	1.19, 0.91 to 1.55	.201
vs. Grade D or E	571	383 (67.1)	188 (32.9)		
**Personal diagnosis of cancer**					
Yes	98	49 (50.0)	49 (50.0)	1.10, 0.68 to 1.80	.690
vs. No	1,790	1,046 (58.4)	744 (41.6)		
**Knows someone with cancer**					
Yes	1,113	562 (50.5)	551 (49.5)	1.50, 1.19 to 1.89	**.001**
vs. No or don't know	775	533 (68.8)	242 (31.2)		
**Previously read a screening leaflet**					
Yes	1,058	535 (50.6)	523 (49.4)	1.27, 0.97 to 1.66	.089
vs. No or not sure	830	560 (67.5)	270 (32.5)		
**Previously read an NHS screening website**					
Yes	324	125 (38.6)	199 (61.4)	1.45, 1.07 to 1.97	**.016**
vs. No or not sure	1,564	970 (62.0)	594 (38.0)		
**Discussed screening with doctor/nurse**					
Yes	624	276 (44.2)	348 (55.8)	1.34, 1.03 to 1.74	**.030**
vs. No	1,264	819 (64.8)	445 (35.2)		
**Previously read or heard similar information**					
Yes	662	266 (40.2)	396 (59.8)	2.30, 1.81 to 2.94	**<.0005**
vs. No or not sure	1,226	829 (67.6)	397 (32.4)		
**Endorsed any term(s) as making sense**					
Yes	1,051	426 (40.5)	625 (59.5)	4.92, 3.94 to 6.17	**<.0005**
No	837	669 (79.9)	168 (20.1)		
**Perceived clarity of information**					Overall: .534
Extremely clear	170	87 (51.2)	83 (48.8)	0.85, 0.52 to 1.40	.525
Very clear	636	339 (53.3)	297 (46.7)	0.97, 0.65 to 1.43	.860
Moderately clear	685	424 (61.9)	261 (38.1)	0.78, 0.53 to 1.16	.225
Slightly clear	206	130 (63.1)	76 (36.9)	0.92, 0.57 to 1.49	.745
vs. Not at all clear	191	115 (60.2)	76 (39.8)		
		**Mean (SD)**			
**Age (in years)**	1,888	43.8 (15.7)	43.3 (15.8)	0.99, 0.98 to 1.00	**.003**

N.B. Adjusted ORs and 95% CIs are relative to a stated reference category except age, which is per unit increase; p-values <.05 are in bold; all predictor variables are included in the model

For peer review only

Table 4 – Number of participants (and percentages and 95% CIs) endorsing i) each number of possible terms and ii) each specific term as making sense to them as a label

		Number of terms endorsed as making sense as a way of describing the text: n, %, 95% CIs							
**Screening information**	Total	0	1	2	3	4	5	6	7
Breast	n (%)	429 (43.5)	76 (7.7)	86 (8.7)	74 (7.5)	67 (6.8)	58 (5.9)	42 (4.3)	155 (15.7)
n=987	95% CI	40.4 to 46.6	6.2 to 9.5	7.1 to 10.6	6.0 to 9.3	5.3 to 8.5	4.5 to 7.5	3.1 to 5.7	13.5 to 18.1
Prostate	n (%)	408 (45.3)	59 (6.5)	73 (8.1)	68 (7.5)	66 (7.3)	48 (5.3)	37 (4.1)	142 (15.8)
n=901	95% CI	42.1 to 48.5	5.1 to 8.3	6.5 to 10.0	6.0 to 9.4	5.8 to 9.2	4.0 to 6.9	3.0 to 5.6	13.5 to 18.2
Total	n (%)	837 (44.3)	135 (7.2)	159 (8.4)	142 (7.5)	133 (7.0)	106 (5.6)	79 (4.2)	297 (15.7)
N=1,888	95% CI	42.1 to 46.6	6.1 to 8.4	7.2 to 9.7	6.4 to 8.8	6.0 to 8.3	4.6 to 6.7	3.4 to 5.2	14.1 to 17.4

		"Which terms make sense as a way of describing this risk of a screening test?": n, %, 95% CIs						
**Screening information**	Total	Overdiagnosis	Overdetection	Unnecessary diagnosis	Overtreatment	Unnecessary treatment	False positive diagnosis	False positive test results
Breast	n (%)	334 (33.8)	275 (27.9)	349 (35.4)	336 (34.0)	409 (41.4)	324 (32.8)	338 (34.2)
n=987	95% CI	30.9 to 36.8	25.1 to 30.7	32.4 to 38.4	31.1 to 37.0	38.4 to 44.5	30.0 to 35.8	31.3 to 37.2
Prostate	n (%)	291 (32.3)	247 (27.4)	307 (34.1)	280 (31.1)	354 (39.3)	320 (35.5)	330 (36.6)
n=901	95% CI	29.3 to 35.4	24.6 to 30.4	31.0 to 37.2	28.1 to 34.2	36.1 to 42.5	32.4 to 38.7	33.5 to 39.8
Total	n (%)	625 (33.1)	522 (27.6)	656 (34.7)	616 (32.6)	763 (40.4)	644 (34.1)	668 (35.4)
N=1,888	95% CI	31.0 to 35.3	25.7 to 29.7	32.6 to 36.9	30.5 to 34.8	38.2 to 42.6	32.0 to 36.3	33.2 to 37.6

For peer review only

Table 5 – Endorsed one or more vs. no terms as making sense: descriptive statistics, adjusted ORs, 95% CIs, and p-values for variables in the multivariable binary logistic regression model

Characteristic	Total (N=1,888)	Endorsed no vs. at least one term as making sense: n (%) — No term (n=837; 44.3%)	At least one term (n=1,051; 55.7%)	Adjusted OR, 95% CI — At least one term endorsed (vs. No terms endorsed)	p-value
**Overdiagnosis information**					
Breast screening text	987	429 (43.5)	558 (56.5)	1.16, 0.95 to 1.42	.148
vs. Prostate screening text	901	408 (45.3)	493 (54.7)		
**Gender**					
Male	879	380 (43.2)	499 (56.8)	1.40, 1.13 to 1.74	**.002**
vs. Female	1,009	457 (45.3)	552 (54.7)		
**Ethnicity**					
White British	1,432	592 (41.3)	840 (58.7)	1.25, 0.97 to 1.61	.080
vs. Other ethnic groups	456	245 (53.7)	211 (46.3)		
**Marital status**					
Married or living as a couple	1,133	500 (44.1)	633 (55.9)	1.05, 0.85 to 1.30	.671
vs. Single, widowed, divorced, or separated	755	337 (44.6)	418 (55.4)		
**Highest level of education**					Overall: .556
Other levels	199	88 (44.2)	111 (55.8)	1.21, 0.80 to 1.82	.374
Approximately Level 4	507	190 (37.5)	317 (62.5)	1.27, 0.88 to 1.84	.204
Approximately Level 1, 2, or 3	890	396 (44.5)	494 (55.5)	1.24, 0.91 to 1.70	.169
vs. No formal qualifications or don't know	292	163 (55.8)	129 (44.2)		
**Social class grade**					Overall: .574
Grade A or B	386	137 (35.5)	249 (64.5)	1.09, 0.78 to 1.51	.617
Grade C1 or C2	931	420 (45.1)	511 (54.9)	0.94, 0.74 to 1.19	.604
vs. Grade D or E	571	280 (49.0)	291 (51.0)		
**Personal diagnosis of cancer**					
Yes	98	37 (37.8)	61 (62.2)	1.08, 0.68 to 1.74	.741
vs. No	1,790	800 (44.7)	990 (55.3)		
**Knows someone with cancer**					
Yes	1,113	427 (38.4)	686 (61.6)	1.35, 1.09 to 1.68	**.005**
vs. No or don't know	775	410 (52.9)	365 (47.1)		
**Previously read a screening leaflet**					
Yes	1,058	416 (39.3)	642 (60.7)	1.18, 0.91 to 1.52	.206
vs. No or not sure	830	421 (50.7)	409 (49.3)		
**Previously read an NHS screening website**					
Yes	324	109 (33.6)	215 (66.4)	1.01, 0.75 to 1.37	.955
vs. No or not sure	1,564	728 (46.5)	836 (53.5)		
**Discussed screening with doctor/nurse**					
Yes	624	217 (34.8)	407 (65.2)	1.25, 0.97 to 1.61	.091
vs. No	1,264	620 (49.1)	644 (50.9)		
**Previously read or heard similar information**					
Yes	662	224 (33.8)	438 (66.2)	1.18, 0.93 to 1.50	.185
vs. No or not sure	1,226	613 (50.0)	613 (50.0)		
**Previously encountered any term(s)**					
Yes	1,051	168 (21.2)	625 (78.8)	4.88, 3.89 to 6.11	**<.0005**
No	837	669 (61.1)	426 (38.9)		
**Perceived clarity of information**					Overall: .148
Extremely clear	170	67 (39.4)	103 (60.6)	1.36, 0.85 to 2.17	.204
Very clear	636	255 (40.1)	381 (59.9)	1.38, 0.96 to 1.98	.083
Moderately clear	685	308 (45.0)	377 (55.0)	1.39, 0.97 to 1.98	.073
Slightly clear	206	109 (52.9)	97 (47.1)	0.99, 0.64 to 1.53	.953
vs. Not at all clear	191	98 (51.3)	93 (48.7)		
		**Mean (SD)**			
**Age (in years)**	1,888	43.4 (15.6)	43.7 (15.8)	1.00, 0.99 to 1.00	.262

N.B. Adjusted ORs and 95% CIs are relative to a stated reference category except age, which is per unit increase; p-values <.05 are in bold; all predictor variables are included in the model

For peer review only - http://bmjopen.bmj.com/site/about/guidelines.xhtml

DISCUSSION

This study aimed to address a gap in the literature regarding how best to communicate the issue of overdiagnosis (8). We investigated whether people are familiar with terms used to define overdiagnosis or related concepts, and whether these made sense as labels for either of two widely used descriptions. We found that a majority of participants (58.0%) had never encountered any of the assessed terms before and a large proportion (44.3%) did not rate any as making sense as a suitable label. In addition, no specific term(s) emerged as being notably more familiar or applicable.

An earlier similar study found that only 30.0% of the UK general public (aged 50-70) had previously encountered the specific term, 'overdiagnosis' (9), which is broadly comparable to the 21.8% who reported having encountered it in this study. Our findings are also consistent with those of a previous study carried out as part of the redesign of the information materials for the NHS Breast Screening Programme (20). Women were interviewed with the aim of finding ways to communicate the concept of overdiagnosis: participants generally struggled to understand it, regardless of whether it was labelled as 'overdiagnosis' or 'overtreatment'.

Terminology can have a role in transferring information to lay individuals as part of a description of technical concepts (11,21). However, the main implication of our results is that there may currently only be limited value in using any of the labels that we tested. Terms used were neither familiar nor rated as particularly relevant in this large, diverse sample, meaning that they may be confusing, unintuitive, and hard for people to memorise and recall. Even the most technically appropriate terms (e.g. 'overdiagnosis' and 'overdetection') were not endorsed as making sense more frequently than suggested alternative terminology (e.g. 'unnecessary diagnosis) or more inaccurate and potentially problematic terminology (e.g. 'false positive

For peer review only

diagnosis'). To some extent, the currently limited familiarity and low levels of endorsement supports UK policy decisions not to use a specific term in the revised breast screening information leaflet (20). Furthermore, shortly before the start of recruitment, an updated NHS information leaflet regarding PSA screening was published, with additional detail on overdiagnosis and overtreatment (22); this also omitted any specific descriptive label.

A potential explanation for the low proportions of participants endorsing each term comes from a recent survey in the United States. This used a similar design to the present study in which women aged 35-55 years were recruited to respond to brief descriptions of overdiagnosis and overtreatment. Only a small minority of participants (approximately 15-20%) endorsed messages as either believable and something with which they agreed (23). To some extent, this is consistent with qualitative research showing that the concept is often surprising and confusing to lay people (6, 7). Limited acceptance of the description of overdiagnosis itself may have resulted in participants disengaging with the concept and hence not rating any of the available terms as appropriate. It may be relevant that both sets of descriptions were presented outside of contexts in which they would normally be encountered (e.g. the original screening information leaflets from which they were adapted). Both comprehension and acceptance may be different (either better or worse) in the presence of additional detail (15), either from a full leaflet or as part of a discussion with a clinician. This warrants further research.

One unexpected finding was that the second most common number of terms that were familiar or endorsed was all seven (12.3% and 15.7%, respectively). This may be due to the differences between terms being subtle: members of the public may not recall exactly which terms they had encountered but felt that broader concepts like diagnosis and treatment were familiar.

For peer review only

We found that participants with a lower level of education, who were not White British, were older, or (possibly) were from a lower social class grade were less likely to have previously encountered any term(s), suggesting that these groups may be particularly poorly served by the use of any specific terminology. Unsurprisingly, those who had previous exposure to cancer (i.e. by knowing someone with a diagnosis) and those who had encountered potentially relevant information (e.g. those who had previously discussed screening with a doctor or nurse) were more likely to have encountered any term(s). In some respects, this latter finding was surprising because although medical staff might be expected to discuss overdiagnosis with patients, there is evidence that the topic is explained only rarely (24). Those who knew someone with cancer were also more likely to endorse at least one term as making sense. It is unclear why males were more likely to endorse term(s) as making sense. This may warrant further research (possible explanations may include a degree of over-confidence in men or under-confidence in women), although this should be considered in the context of small absolute differences observed here (56.8% vs. 54.7% endorsing at least one term).

Continuing communication efforts mean that these results may change over time: it is plausible that ongoing campaigns (e.g. 3-5) will result in the public becoming increasingly familiar with the concept and terminology, having a better understanding of the differences between similar terms (e.g. seeing ‘false positives’ as distinct from ‘overdiagnosis’), and being more likely to endorse particular terms as applicable. However, present attempts to communicate overdiagnosis (e.g. as part of screening invitations or mainstream media stories) may be more effective if explicit descriptions are used, such as adapted forms of information from the Breast Screening Programme. This has undergone an extensive design process (20) and is more likely to be rated as clearer than the equivalent from the Prostate Cancer Risk Management Programme. However, fewer than half of participants (46.6%) rated it as very or extremely clear,

For peer review only

suggesting that future research could be used to improve it further (e.g. by providing additional detail; 15).

This study has limitations. The data collection method used by the survey company meant that demographic characteristics used to ensure population-representativeness were not exhaustive (e.g. they omitted ethnicity and education) and response rates were not available. The list of terms tested was also not exhaustive; there may be suitable terminology that we did not assess. However, we found very similar levels of endorsement for terms that used several variations on the themes of diagnosis, treatment, and false positives, providing some evidence that any superior terms would have to be quite different.

Conclusions

Alternative terminology to ‘overdiagnosis’ may help increase awareness and understanding of the concept among individuals who may face healthcare decisions that would put them at risk. These results suggest that, at present, using specific terms would have limited benefits and may be less well suited to particular groups (e.g. less educated or non-White British individuals). It may currently be more effective to refer to the concept via more explicit descriptions. Previous research indicates that information from the NHS Breast Screening Programme may be a relatively effective template for this purpose. However, future research could explore scope for improvement.

Contributors: AG, CR, and JW conceived and designed the study. AG analysed the data. AG, CR, and JW participated in the interpretation of results. AG, CR, and JW drafted the manuscript, participated in critical revision, and approved the final version.

For peer review only

Funding: This work was supported by a programme grant from Cancer Research UK awarded to Prof Jane Wardle [C1418/A14134]. Dr Jo Waller is supported by a Career Development Fellowship from Cancer Research UK [C7492/A17219]. Cancer Research UK was not involved in the design of this study; the collection, analysis, or interpretation of the results; in the writing of the manuscript; or in the decision to submit for publication.

Competing interests: None declared.

Patient consent: Obtained.

Data sharing: No additional data are available.

REFERENCES

1. Heath I. Overdiagnosis: when good intentions meet vested interests — an essay by Iona Heath. Br Med J. 2013;347:f6361.

2. Moynihan R, Doust J, Henry D. Preventing overdiagnosis: how to stop harming he healthy. Br Med J. 2012;344:e3502.

3. Glasziou P, Moynihan R, Richards T. Godlee F. Too much medicine; too little care. Br Med J. 2013;347:f4247.

4. Malhotra A, Maughan D, Ansell J, et al. Choosing Wisely in the UK: the Academy of Medical Royal Colleges' initiative to reduce the harms of too much medicine. Br Med J. 2015;350:h2308.

5. Calderwood C. Chief Medical Officer's annual report 2014-15. 2016. http://www.gov.scot/Publications/2016/01/3745/0

For peer review only

6. Hersch J, Jansen J, Barratt A, et al. Women's views on overdiagnosis in breast cancer screening: a qualitative study. Br Med J. 2013;346:f158.

7. Waller J, Douglas E, Whitaker KL, Wardle J. Women's responses to information about overdiagnosis in the UK breast cancer screening programme: a qualitative study. BMJ Open. 2013;3:e002703.

8. McCaffery KJ, Jansen J, Scherer LD, et al. Walking the tightrope: communicating overdiagnosis in modern healthcare. Br Med J. 2016;352:i348.

9. Ghanouni A, Meisel SF, Renzi C, Wardle J, Waller J. Public definitions of the term 'overdiagnosis' in the UK. BMJ Open. 2016;e010723.

10. Moynihan R, Nickel B, Hersch J, et al. What do you think overdiagnosis means? A qualitative analysis of responses from a national community survey of Australians. BMJ Open. 2015;5:e007436.

11. Hersch J, Jansen J, Barratt A, et al. Overdetection in breast cancer screening: development and preliminary evaluation of a decision aid. BMJ Open. 2014;4:e006016.

12. Moynihan R, Nickel B, Hersch J, et al. Public opinions about overdiagnosis: a national community survey. PLoS One. 2015;10:e0125165.

13. NHS Breast Screening Programme. NHS Breast Screening: Helping you decide. 2013. Available from: https://www.gov.uk/government/uploads/system/uploads/attachment_data/file/440798/nhsbsp.pdf

14. Burford D, Austoker J, Kirby M. PSA (prostate specific antigen) testing for prostate

For peer review only

cancer. An information sheet for men considering a PSA test. 2009.

15. Ghanouni A, Renzi C, McBride E, Waller J. Comparing perceived clarity of information on overdiagnosis used for breast and prostate cancer screening in England: an experimental survey. BMJ Open. 2017;7:e015955.

16. National Readership Survey. Social grade. http://www.nrs.co.uk/nrs-print/lifestyle-and-classification-data/social-grade/ Accessed: 1st December 2017.

17. Weinstein ND. The precaution adoption process. Heal Psychol. 1988;7:355–86.

18. Willis GB , Artino AR . What do our respondents think we're asking? using cognitive interviewing to Improve Medical Education surveys. J Grad Med Educ 2013;5:353–6.

19. Office of National Statistics. 2011 Census: Key Statistics for England and Wales, March 2011. 2012 http://www.ons.gov.uk/ons/rel/census/2011-census/key-statistics-for-local-authorities-in-england-and-wales/stb-2011-census-key-statistics-for-england-and-wales.html (accessed 28 Nov 2016).

20. Forbes LJ, Ramirez AJ, Expert group on Information about Breast Screening. Offering informed choice about breast screening. J Med Screen. 2014;21:194–200.

21. Fage-Butler AM, Nisbeth Jensen M. Medical terminology in online patient-patient communication: evidence of high health literacy? Heal Expect. 2016;19:643–53.

22. Public Health England. PSA testing and prostate cancer: advice for well men aged 50 and over. 2016. Available from: https://www.gov.uk/government/uploads/system/uploads/attachment_data/file/509191/Patient_info_sheet.pdf

For peer review only

23. Nagler RH, Fowler EF, Gollust SE. Women's awareness of and responses to messages about breast cancer overdiagnosis and overtreatment: Results from a 2016 national survey. Med Care. 2017;55:879-85.

24. Wegwarth O, Gigerenzer G. Less is more: overdiagnosis and overtreatment: evaluation of what physicians tell their patients about screening harms. JAMA Intern Med. 2013;173:2086-7.

For peer review only
